# A genome-wide microRNA profiling indicates miR-424-5p and miR-503-5p as regulators of ALK expression in neuroblastoma

**DOI:** 10.18632/oncotarget.17033

**Published:** 2017-04-11

**Authors:** Marilena De Mariano, Sara Stigliani, Stefano Moretti, Federica Parodi, Michela Croce, Cinzia Bernardi, Aldo Pagano, Gian Paolo Tonini, Silvano Ferrini, Luca Longo

**Affiliations:** ^1^ UOC Bioterapie, Dipartimento di Terapie Oncologiche Integrate, IRCCS AOU San Martino-IST, Istituto Nazionale per la Ricerca sul Cancro, Genoa, Italy; ^2^ UOS Fisiopatologia della Riproduzione Umana, Dipartimento di Chirurgia Generale, Specialistica ed Oncologica, IRCCS AOU San Martino-IST, Istituto Nazionale per la Ricerca sul Cancro, Genoa, Italy; ^3^ Université Paris-Dauphine, PSL Research University, CNRS, Department UMR [7243], LAMSADE, Paris, France; ^4^ Centre of Excellence for Biomedical Research (CEBR), University of Genoa, Genoa, Italy; ^5^ Dipartimento di Terapie Oncologiche Integrate, IRCCS AOU San Martino-IST, Istituto Nazionale per la Ricerca sul Cancro, Genoa, Italy; ^6^ Department of Experimental Medicine (DIMES), University of Genoa, Genoa, Italy; ^7^ Neuroblastoma Laboratory, Pediatric Research Institute, Città della Speranza, Padua, Italy

**Keywords:** neuroblastoma, microRNA, ALK, miR-424-5p, miR-503-5p

## Abstract

The discovery of missense mutations of *ALK* gene identified this receptor tyrosine kinase as a therapeutic target in neuroblastoma (NB). Moreover, a high level of ALK protein has been associated with metastatic NB cases and with a worse prognosis, suggesting that also ALK overexpression is involved in NB tumorigenesis. Since miRNAs play key roles in the regulation of gene expression we aimed at identifying those miRNAs that can regulate ALK in NB. We therefore analyzed the genome-wide expression profile of miRNAs in two sample sets of 16 NB cell lines and 22 NB samples by using miRNA microarrays. Both sample sets were then divided into two subgroups showing high (ALK+) or low/absent (ALK-) expression of ALK. Results showed a down-regulation of 30 and 23 miRNAs (p-value <0.05) in the ALK+ group in NB cell lines and samples, respectively. Validation analysis indicated that miR-424-5p and miR-503-5p, belonging to the same cluster, were differentially expressed in both NB cell lines and tumor samples. Although only miR-424-5p showed a direct binding to ALK 3′-UTR, both miRNAs led to a remarkable decreasing of ALK protein as well as to the inhibition of cell viability in ALK+ NB cell lines. In conclusion, our data indicate that both miR-424-5p and miR-503-5p are involved in regulating ALK expression in NB, either by directly targeting ALK receptor or indirectly, and may thus serve as potential therapeutic tools in ALK dependent NBs.

## INTRODUCTION

Neuroblastoma (NB) is a neural crest-derived pediatric cancer that shows a peculiar heterogeneity in its clinical presentation and outcome as well as in the molecular mechanisms that underlie its genetic predisposition and aggressiveness [1, 2]. Over the years many studies have contributed to shed light on several non-random somatic alterations that characterize NB [1], including *MYCN* proto-oncogene amplification, which is associated with aggressive clinical disease [3–5].

The Anaplastic Lymphoma Kinase (ALK) is a trans-membrane receptor tyrosine kinase (RTK), belonging to the insulin receptor superfamily of RTKs. The expression of ALK is restricted to the developing nervous system and is supposed to play a role in the regulation of neuronal differentiation [6]. ALK catalytic domain was originally identified in the t(2;5)(p23;q35) chromosomal translocation that produces the oncogenic fusion of the amino terminus of nucleophosmin (NPM) to the catalytic intracellular domain of ALK [7]. This rearrangement occurs in most of the Anaplastic Large Cell Lymphomas (ALCL), which mainly affect children and young adults [7]. Since then, ALK has been found to be involved in several other cancers, mainly as a consequence of different chromosomal translocations such as those reported in inflammatory myofibroblastic tumors [8], non-small cell lung cancer [9] and diffuse large B-cell lymphomas [10]. In 2008 *ALK* emerged also as a major predisposition gene in NB, paving the way for new target-based therapeutical approaches for this tumor [11–14]. Indeed, several studies led to the identification of both germline and somatically acquired activating missense point mutations affecting the tyrosine kinase domain of ALK in about half of the families with recurrence of NB and 8-10% of sporadic NB cases [11–14], although this percentage increases significantly in the relapsed patient population [15, 16]. Among more than 20 different ALK mutations discovered so far, F1174, R1275 and F1245 are the three most frequent mutated codons, accounting for about 87% of all mutations affecting this RTK. They have been classified as gain-of-function mutations and demonstrated to have transforming capacities [17]. Moreover, about 4% of high-risk NB patients show amplification of the *ALK* genomic locus, as well [18]. Therefore, great efforts are now aimed at pharmacologically inhibiting ALK activity in NB cells by taking advantage of small molecule inhibitors such as crizotinib, an FDA-approved anticancer drug that exhibited efficacy in non-small cell lung cancers harboring *ALK* translocations [19]. Conversely, crizotinib showed limited activity in NB patients with ALK-activating mutations [20] that revealed differential sensitivity to ALK inhibitors in both preclinical and clinical studies [18, 21].

ALK expression in NB was first described by Lamant and colleagues [22] and more recently we and others reported that wild type ALK is frequently overexpressed in metastatic NB and that, similarly to activating ALK mutations, its overexpression is correlated with poor clinical outcome [23, 24]. Hence, it has been suggested that in addition to activating mutations also an aberrant level of wild type ALK expression could be involved in NB oncogenesis and progression, although it probably should reach a critical threshold for triggering ALK activation [23]. Indeed, primary NBs with *ALK* mutations were reported to invariably exhibit high levels of ALK expression [24], and NBs with an expression of wild type ALK comparable to that of ALK mutated tumors showed a similar poor outcome [24]. Notably, wild type ALK expression in the neural crest progenitor cells JoMa1 can drive the formation of malignant tumors in nude mice [25]. Altogether, these results suggested that the inhibition of either mutated or wild type ALK expression may exert beneficial effects in NB patients.

Protein expression can be regulated by microRNAs (miRNAs), which are short, single-stranded RNA molecules [26], and constitute a family of highly conserved non-coding small RNAs. Indeed, miRNAs down-regulate the expression of specific proteins at the post-transcriptional level, mainly by targeting the 3′-untranslated region (3′-UTR) of their target mRNAs. Therefore, miRNAs play key roles in various cellular processes in both animals and human diseases [26]. In human cancers, miRNAs can function as oncogenes or tumor suppressors and their expression profile may correlate to prognosis, diagnosis and response to treatment [27]. The role of miRNAs in human cancers was initially demonstrated in chronic lymphocytic leukemia, where a loss of miR-15a and miR-16-1 due to the 13q14 deletion was found in over half of cases [28]. Since then, the deregulation of miRNAs expression has been demonstrated in other types of cancers, including NB [29–34]. Although a large number of miRNAs have been identified to date, the role of the vast majority of them in tumorigenesis remains to be determined. This work is aimed at identifying those miRNAs that can specifically regulate ALK protein levels. Indeed, in our view the design of novel therapeutic strategies that lead to the modulation of miRNAs activity, and consequently to the inhibition of ALK expression in NB, might constitute a novel approach for the treatment of NB.

## RESULTS

### Determination of ALK+ and ALK- subgroups in NB cell lines

**Figure 1:**
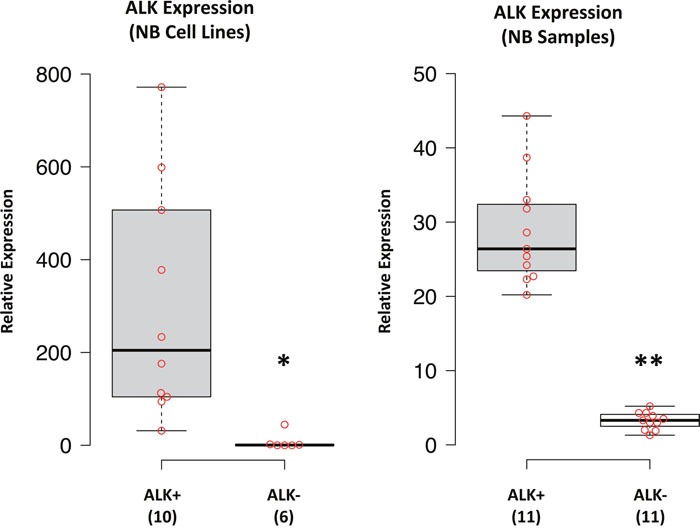
ALK expression in NB cell lines and samples *ALK* mRNA expression was performed by qPCR and data from NB cell lines and tumor samples were box-plotted (beeswarm box plot) using BoxPlotR, a web-tool for generation of box plots publicly available at http://boxplot.tyerslab.com. Numbers in the Y-axis represents the fold change in expression with respect to the less expressed sample, which was set as 1. Histograms reporting expression results for each sample from NB cell lines and tumors are shown in Supplementary Figures 1 and 2, respectively. p-value < 0.05 (*); p-value < 0.01 (**).

To identify miRNAs differentially expressed between ALK+ and ALK- NB cell lines we carried out analyses in a set of NB cell lines to determine both mRNA and protein ALK expression. We thus identified two subgroups of cell lines showing either high ALK expression (ALK+) or low/absent ALK expression (ALK-) (Figure 1). The expression of ALK protein was detected in 10 out of 16 NB cell lines (Supplementary Figure 1B) that were thus considered as belonging to the ALK+ subgroup. Although SKNBE2C cell line showed a low amount of ALK mRNA (Supplementary Figure 1A), we could not detect any protein expression (Supplementary Figure 1B). Therefore, we considered SKNBE2C as belonging to the ALK- subgroup, which consisted of 6 NB cell lines.

### Determination of ALK+ and ALK- subgroups in NB tumor samples

Similarly, to identify miRNAs differentially expressed between ALK+ and ALK- NB tumors we first analyzed ALK mRNA expression in a set of tumor samples to determine subgroups of NBs expressing low or high levels of ALK. Since the amount of frozen tumor tissue was insufficient to perform protein analysis, we grouped NB tumor samples on the basis of their different ALK mRNA expression levels, thus identifying an ALK+ and an ALK- subgroup, each consisting of 11 samples (Figure 1, Supplementary Figure 2). These 22 NB tumor samples were derived from a selection of 120 frozen NB specimens that were randomly chosen from our NB tissue bank, and considered eligible for RNA extraction only if tumor cell content reached 70%. After quality control and determination of RNA concentrations, 75 samples resulted suitable for subsequent analyses. Moreover, in order to rule out a possible bias in both miRNAs and ALK expression we excluded 17 samples that showed either *MYCN* amplification or high MYCN expression (Supplementary Figure 3A), although high levels of MYCN mRNA did not significantly influence those of ALK in our sample set (Supplementary Figure 4). Last, from the remaining 58 *MYCN* single copy NB samples we defined the two ALK+ and ALK- subgroups. To maximize the difference in ALK expression between the two subgroups, we selected the 11 samples that showed the highest and the lowest expression of ALK (Figure 1). MYCN mRNA expression in both subgroups was evenly distributed (Supplementary Figure 3B).

### Identification of differentially expressed miRNAs in ALK+ and ALK- NB cell lines and tumor samples

**Figure 2:**
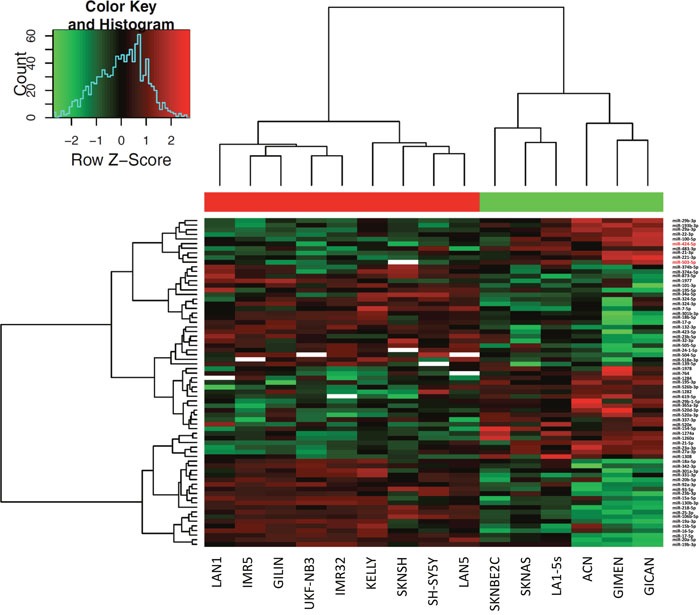
Hierarchical clustering of differentially expressed miRNAs in NB cell lines Differential expression analysis between ALK+ and ALK- categories was carried out using Student’s t-test (unpaired, two-tailed, unequal variance). SKNBE2 cell line was removed from the analysis for low quality hybridization. 71 miRNAs showed raw p-value < 0.05. miR-424-5p and miR-503-5p are highlighted with a red font.

**Figure 3:**
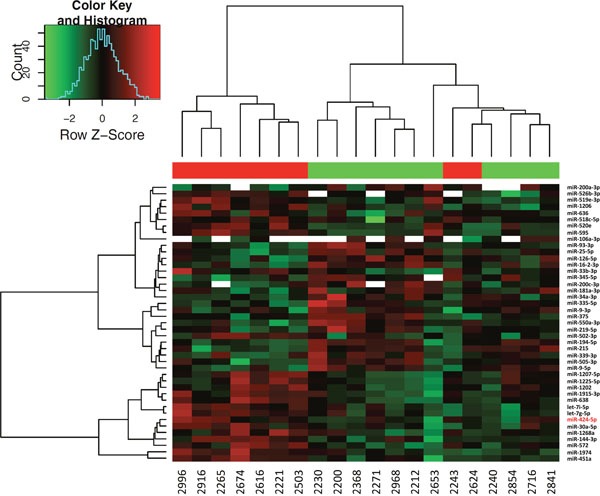
Hierarchical clustering of differentially expressed miRNAs in NB tumor samples Differential expression analysis between ALK+ and ALK- categories was carried out using Student’s t-test (unpaired, two-tailed, unequal variance). Samples 2731 and 2347 were removed from the analysis for low quality hybridization. 43 miRNAs showed raw p-value < 0.05. miR-424-5p is highlighted with a red font.

**Figure 4:**
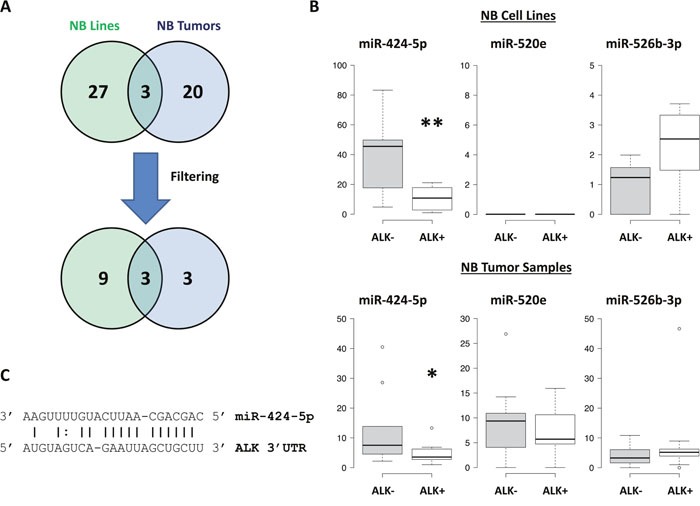
Validation of candidate miRNAs **(A)** Venn diagram representing the number of miRNAs that were found to be down-regulated in ALK+ group (or up-regulated in ALK- group). Filtering criteria for prioritizing miRNAs for validation and functional analyses are described in the results. **(B)** Validation analysis of miRNAs expression was performed by qPCR and data were box-plotted using BoxPlotR. p-value < 0.05 (*); p-value < 0.01 (**). **(C)** miR-424-5p/*ALK* 3′-UTR alignment as predicted by MicroRNA.org. Sequence starts at 321 bp of *ALK* 3′-UTR. mirSVR score:-0.9220; PhastCons score: 0.5638.

MiRNAs expression analysis was carried out in 16 NB cell lines and 22 tumor samples by using Human microRNA Microarray (Agilent Technologies), consisting in a set of probes for 866 human miRNAs. After filtering, condensing by replicates average and controlling spots removal, the quantitative analysis provided an expression dataset composed by 388 and 675 miRNAs in NB cell lines and NB samples, respectively. We identified two relevant sets of 71 and 43 miRNAs, which were differentially expressed in ALK+ and ALK- subgroups in NB cell lines and NB samples, respectively (raw p-values < 0.05), and then made a hierarchical clustering to detect similarity relationships in miRNAs log2-transformed expressions (Figure 2, Figure 3). Overall, 30/71 miRNAs in NB cell lines and 23/43 in NB samples were down-regulated in the ALK+ subgroup. Interestingly, three miRNAs (miR-424-5p, miR-520e, and miR-526b-3p) emerged as candidates from both analyses (Figure 4A). We then considered for validation analysis by qPCR 12/30 miRNAs from cell lines (Table 1) and 6/23 from tumor samples (Table 2) for a total number of 15 miRNAs, as three were included in both sample sets. The other 12 candidate miRNAs were selected if they matched one or more of the following criteria: i) in silico prediction of potential *ALK* 3′-UTR targeting; ii) fold change in miRNAs expression > 2; iii) supporting data from literature for an involvement of the candidate miRNA with either ALK, NB or cancer in general.

**Table 1:** Candidate miRNAs from microarray analysis of NB cell lines

miRNA	ALK- group	ALK+ group	rawp-value	Fold change
hsa-miR-526b-3p	1.92	0.27	0.042	3.14
hsa-miR-424-5p	8.02	6.72	0.008	2.48
hsa-miR-520e	2.86	2.13	0.032	1.65
hsa-miR-100-5p	7.87	3.59	0.019	19.45
hsa-miR-29b-3p	8.17	4.12	0.04	16.65
hsa-miR-29a-3p	9.48	5.46	0.009	16.20
hsa-miR-503-5p	5.68	2.95	0.022	6.65
hsa-miR-221-3p	5.56	2.88	0.015	6.41
hsa-miR-21-5p	11.31	8.73	0.002	5.98
hsa-miR-22-3p	7.82	5.60	0.007	4.67
hsa-miR-29b-1-5p	4.13	2.64	0.014	2.81
hsa-miR-23a-3p	7.93	6.68	0.016	2.37

The table reports data only for miRNAs selected for validation analysis. Expression values reported in the ALK+ and ALK- columns were log_2_-transformed and normalized.

**Table 2:** Candidate miRNAs from microarray analysis of NB tumor samples

miRNA	ALK- group	ALK+ group	rawp-value	Fold change
hsa-miR-424-5p	6.54	4.72	0.017	3.51
hsa-miR-526b-3p	3.24	2.55	0.037	1.62
hsa-miR-520e	3.56	2.97	0.01	1.49
hsa-miR-451a	14.75	11.74	0.011	8.08
hsa-let-7g-5p	11.86	10.41	0.036	2.74
hsa-let-7i-5p	11.69	10.30	0.042	2.62

The table reports data only for miRNAs selected for validation analysis. Expression values reported in the ALK+ and ALK- columns were log_2_-transformed and normalized.

### Validation of candidate miRNAs by qPCR

Validation analysis of the 15 candidate miRNAs was performed by qPCR on both sets of samples employed for miRNAs expression screening, which included 16 NB cell lines and 22 NB tumor samples. Of the three miRNAs identified by both genome-wide screenings only miR-424-5p was validated in both NB cell lines and tumor samples (Figure 4B). Conversely, miR-21-5p, miR-22-3p, miR-23a-3p, miR-29a-3p, miR-29b-3p, miR-29b-1-5p, miR-100-5p and miR-221-3p were validated in NB cell lines only (i.e. the sample set from which they emerged as candidates) (Supplementary Figure 5), whereas let-7g-5p and miR-451a were validated in tumor samples only (i.e. the sample set from which they emerged as candidates) (Supplementary Figure 6). Notably, miR-503-5p, which is a member of the same cluster that includes miR-424-5p, was also found to be differentially expressed in both sets of specimens (Supplementary Figures 5, 6).

### Luciferase assay indicates a direct binding of miR-424-5p to *ALK* 3′-UTR

**Figure 5:**
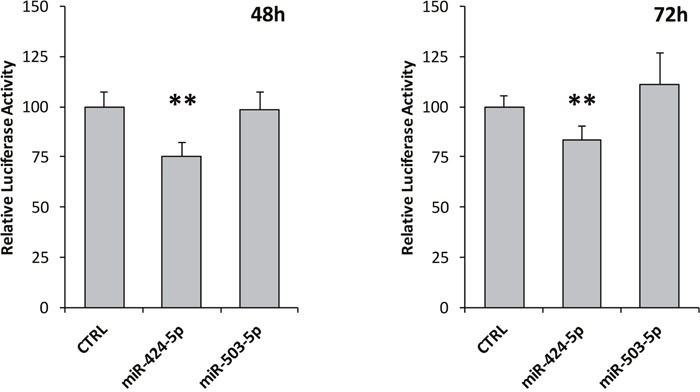
*ALK 3*’-UTR luciferase assay Firefly and Renilla luciferase activities were assessed 48 and 72 hours after transfection with miR-424-5p and miR-503-5p mimics in SKNAS cells. Firefly luciferase was firstly normalized to Renilla luciferase activity, and then normalized to the luciferase activity ratio observed in the empty vector. Negative Control siRNA values were set as 100%. p-value < 0.05 (*); p-value < 0.01 (**).

Target prediction program MiRanda (www.microRNA.org) predicted a potential miRNA binding site at *ALK* 3′-UTR for miR-424-5p (Figure 4C). In order to assess whether miR-424-5p as well as miR-503-5p could regulate luciferase expression, we cloned the whole 450 bp of *ALK* 3′-UTR in the pMIR-REPORT vector, containing a transcriptional unit encoding firefly luciferase. The luciferase reporter vector and the renilla control vector were then transfected in a NB ALK negative cell line, namely SKNAS, together with miR synthetic mimics or a control siRNA. As a matter of fact, after normalization to renilla, the transfection of miR-424-5p mimic led to a reduction of about 24% and 16% of the relative luciferase activity after 48 and 72 hours, respectively, as resulting from a set of three independent experiments (Figure 5). Conversely, miR-503-5p transfections did not exhibit any reduction of luciferase activity (Figure 5).

### Both miR-424-5p and miR-503-5p down-regulate ALK protein expression and limit cell viability in ALK+ NB cell lines

**Figure 6:**
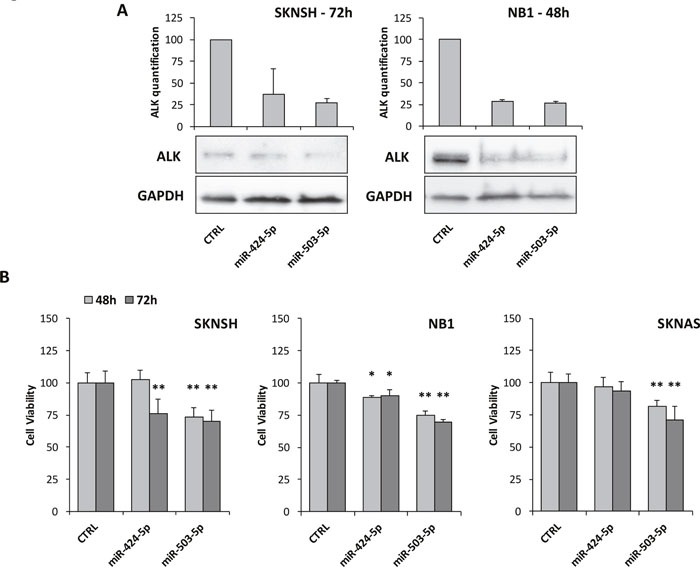
Impairment of ALK expression and cell viability assay **(A)** Western blot analysis of ALK after transient transfection with miR-424-5p and miR-503-5p mimics. SKNSH cells revealed a marked decreasing of ALK after 72 hours. NB1 cells showed a similar ALK reduction after 48 hours, although its expression restored at 72 hours (data not shown). Quantification of bands was performed by ImageJ software and experiments were repeated at least once with consistent results. (B) MTT test to assess cell viability after transient transfection with miR-424-5p and miR-503-5p mimics at 48 and 72 hours in two ALK+ cell lines (SKNSH and NB1) and one ALK- cell line (SKNAS). p-value < 0.05 (*); p-value < 0.01 (**).

In order to demonstrate if miR-424-5p and miR-503-5p might indeed regulate ALK expression, we analyzed two ALK+ NB cell lines (i.e. NB1 and SKNSH) by western blot after transiently transfecting them with either miR-424-5p or miR-503-5p mimics. Although we did not observe a direct binding of miR-503-5p to *ALK* 3′-UTR, results showed that both miRNA mimics led to a remarkable reduction of ALK protein at 48 and 72 hours post transfection in NB1 and SKNSH cell lines, respectively (Figure 6A), indicating that ALK expression can be regulated by both miRNAs. We next performed MTT analysis after miR-424-5p mimic transfection, which revealed a reduction of cell viability in NB1 cells at 48 and 72 hours and in SKNSH cells at 72 hours. No significant impairment of cell number was observed in the ALK- cell line SKNAS (Figure 6B). Similarly, transfection of miR-503-5p led to a significant reduction of cell viability in both NB1 and SKNSH cells at 48 and 72 hours, but also in SKNAS (Figure 6B). Finally, to better investigate the reduction of cell number observed by MTT, we performed cell cycle analysis in ALK+ and ALK- cell lines. Both miR-424-5p and miR-503-5p induced a G1 accumulation in ALK+ NB1 cells but not in SKNSH, although we observed an accumulation of the G1 phase also in SKNAS cells (Supplementary Figure 7).

## DISCUSSION

Since ALK turned up as a key oncogene involved in the tumorigenesis of at least a subset of NB patients, great efforts have been made to develop an efficient system for its targeting, mainly by taking advantage of small molecule inhibitors, such as crizotinib. ALK missense mutations in general, and ALK F1174L in particular, however, proved to confer a relative resistance to this drug as a consequence of an increased ATP-binding affinity of the mutant, an effect that could be surmountable using higher doses of crizotinib or inhibitors with a higher affinity for the protein [35]. To overcome these difficulties, combinational treatments acting simultaneously on multiple targets as for instance that using PI3K/AKT/mTOR pathway inhibitors combined with crizotinib, have been investigated [36]. In addition, a second generation of ALK inhibitors [37–39] or a combined targeting of an ALK inhibitor with liposomal siRNAs [40] have been proposed as better treatment options if compared with crizotinib alone. Aside from activating point mutations, we previously indicated that ALK expression is significantly up-regulated in metastatic NB, suggesting that its overexpression might contribute to the transformation of neuroblastic cells [23, 24]. Here, we aimed to provide novel insights into the molecular mechanisms that control ALK expression in NB by searching for ALK regulatory miRNAs, thus providing novel tools that might be exploited for inhibition of this membrane receptor.

We set out to determine a list of differentially expressed miRNAs that can distinguish between two groups of NBs, showing either high or low/absent expression of ALK, by a genome-wide approach. In the ALK+ subgroup our analysis identified two relevant sets of 30 and 23 down-regulated miRNAs in NB cell lines and samples, respectively. Among these, miR-424-5p emerged as a promising candidate from both miRNA expression profile analyses. Functional studies on a luciferase reporter vector bearing an *ALK* 3′-UTR insert demonstrated that miR-424-5p can, indeed, bind to *ALK* 3′-UTR. Consistently with this observation, we assessed a remarkable down-regulation of endogenous ALK protein after transient transfection of miR-424-5p in two ALK expressing NB cell lines (SKNSH and NB1). In addition, miR-424-5p mediated down-regulation of ALK also led to a statistically significant decrease of cell viability, as assessed by MTT test in these two cell lines, although we cannot exclude that this effect might be due also to other miR-424-5p targets. Validation analysis of candidate miRNAs suggested also miR-503-5p as being significantly deregulated between ALK+ and ALK- NB cell line and tumor subgroups. Interestingly, miR-503-5p is clustered with miR-424-5p. Although we could not demonstrate a direct interaction with *ALK* 3′-UTR for miR-503-5p, we observed a similar result to that obtained with miR-424-5p in its capability to down-regulate ALK protein as well as limit NB cells viability. However, miR-503-5p induced cell viability impairment also in an ALK- cell line, namely SKNAS, suggesting that this miRNA can act through several different targets. Considering that clustered miRNAs may co-operatively act to regulate a same pathway or biological process, our observations suggest that miR-503-5p could also participate to regulate ALK expression, although indirectly. Both miR-424-5p and miR-503-5p have been shown previously to induce G1 cell cycle arrest in acute myeloid leukemia (AML) cell lines [41]. Cell cycle DNA profile in NB cell lines indicated that both miRNAs can induce a G1 accumulation only in ALK+ NB1 cells, but not in the other ALK+ cell line (SKNSH). Particularly, miR-503-5p exerted a broader effect on G1 accumulation in NB1. However, we observed that both miR-424-5p and miR-503-5p induced an accumulation of the G1 phase also in an ALK- cell line (SKNAS), indicating that in this cell line the effect should be mediated by target(s) of these two miRNAs other than ALK (Supplementary Figure 7). Indeed, both miR-424-5p and miR-503-5p, as well as miRNAs in general, have hundreds of targets, including cell cycle regulators [41], and the effects on cell viability, cell death or cell cycle distribution may be mediated by several of their target transcripts.

MiR-424, namely miR-322 in rodents, maps on human chromosome Xq26.3. Together with miR-503 it is co-transcribed as a polycistronic primary transcript and thus belongs to a large cluster that comprises the miR-424(322)-503 gene cluster [41–43]. Several studies have recently reported an important role played by miR-424 and miR-503 in human malignancies. Indeed, miR-424 controls important biological processes often perturbed in cancer such as cell-cycle progression, angiogenesis, cell differentiation and proliferation [44–46]. In this context it has been shown that miR-424 and miR-503 may potentially act as tumor suppressors [47–51] and that are down-regulated in several human tumors, including hepatocellular carcinoma [52, 53], cervical cancer [54], acute myeloid leukemia [55], ovarian cancer [56], colon cancer [57], lung cancer [58], renal cancer [59], osteosarcoma [60], and bladder cancer [61] where miR-424 has a role in tumor invasion and epithelial-mesenchymal transition. Conversely, other studies reported the up-regulation of miR-424 in pancreatic cancer, suggesting that in a specific context it may also act as a tumor promoter [62]. Moreover, overexpression of miR-424 has been shown to suppress proliferation and induce apoptosis of chronic myeloid leukemia cells carrying the Philadelphia chromosome, sensitizing these cells to Imatinib treatment [63]. Therefore, miR-424 has been proposed as a tumor suppressor acting *via* down-regulation of BCR-ABL and its up-regulation may have a therapeutic effect in chronic myeloid leukemia [63]. Aberrant expression of miR-424-5p is also involved in resistance to anoikis and in epithelial-mesenchymal transition during the metastatic process of hepatocellular carcinoma, and its down-regulation significantly contributes to liver cancer progression [64]. Notably, both miR-424-5p and miR-503-5p came out as down-regulated miRNAs between ALK+ and ALK- ALCLs, since they were included in a signature of 56 miRNAs distinguishing ALK+ ALCL, ALK- ALCL and T-cells [65]. In addition to the wide literature underlining the important role of miR-424-5p and miR-503-5p in tumorigenesis, our results indicate now that the deregulation of these two miRNAs is also involved in controlling ALK expression in NB. To our knowledge this is the first genome-wide miRNA profiling that is aimed at identifying ALK regulator miRNAs. Notwithstanding that, a previous study based on in silico predictions identified miR-96 (i.e. miR-96-5p according to the last miRNA nomenclature conventions) to potentially bind to the ALK 3′-UTR and provided evidence that decreases in miR-96 could represent a mechanism underlying the aberrant expression of ALK in cancer cells [66].

In general, deregulation of miRNAs expression contributes to the pathogenesis of most, if not all, human neoplasias and malignant cells show dependence on the expression of miRNAs, so that small RNAs provide important opportunities for the development of novel miRNA-based therapies [67]. Indeed, miRNAs are considered potential diagnostic and therapeutic tools and the use of miRNA mimics or antagomirs is considered a promising strategy to correct gene regulatory networks and reverse the phenotype of cancer cells. The observation of a relative resistance to ALK inhibitors addresses the significance of discovering novel tools to impair ALK activity in NB. Our results indicate now a contributing role of miR-424-5p and miR-503-5p in controlling ALK expression and, thus, these two miRNAs may serve as novel therapeutic tools for those NBs that depend on ALK activation.

## MATERIALS AND METHODS

### NB cell lines and samples

The following 16 NB cell lines were employed in the genome-wide screening: ACN, GICAN, GILIN, GIMEN, LA1-5S, LAN1, LAN5, IMR32, IMR5, KELLY, SH-SY5Y, SKNAS, SKNBE(2), SKNBE2C, SKNSH, UKF-NB3. NB1 is an ALK amplified cell line and was used in functional experiments only. Name, origin and *MYCN* status of all NB cell lines used in this study are listed in Supplementary Table 1. Cell lines were cultured at 37°C and 5% CO_2_ and maintained in RPMI 1640, supplemented with L-glutamine, penicillin/streptomycin, and 10% FBS (Lonza). Tumor specimens were collected at the onset of disease from 120 patients, who were diagnosed with a primary NB between 2002 and 2008, and referred to the Gaslini Children’s Hospital (Genoa, Italy). All analyses involving human specimens were performed according to the Helsinki declaration. Tumor samples included only specimens with a neuroblastic cell content >70%. Biologic information of the 22 NB tumor samples selected for miRNA genome-wide screening are listed in Supplementary Table 2.

### RNA and miRNA extraction

Total RNA containing miRNAs was extracted by miRNeasy Mini kit (Qiagen), including a DNase step, according to the manufacturer’s instructions. The integrity of total RNA and miRNAs was checked by RNA 6000 Nano and Small RNA assays, respectively, on the 2100 Bioanalyzer (Agilent Technologies) and quantification performed by Nanodrop ND-1000 spectrophotometer (Thermo Scientific). Only RNAs with a RIN ≥7 were included in subsequent experiments. One μg of total RNA, containing miRNAs, was retro-transcribed using miScript Reverse Transcription Kit (Qiagen) and miScript HiFlex Buffer in a final volume of 20 μl. A poly(A) tag was added during the reaction and reverse transcription (RT) was performed in presence of both oligo-dT and random primers. The oligo-dT primers had a universal tag sequence on the 5′ end that, with a specific miScript primer (Qiagen) different for each miRNA, allowed amplification in the qPCR step.

### ALK qPCR

Expression of *ALK* mRNA was analyzed in both NB cell lines and samples by using 25 ng of cDNA in two independent experiments, in duplicate, on EpGradient Realplex PCR System (Eppendorf), using RealMasterMix (Eppendorf). We used specific 6FAM labeled TaqMan Assays (Applied Biosystems) for *ALK*, and employed the geNORM Kit (Primerdesign) to choose the best reference genes (*18S*, *GAPDH* and *UBC*). The comparative ΔCt method was then applied, normalizing *ALK* threshold cycle values on the geometric mean of the three housekeeping genes. All qPCR analyses were conducted in accordance to the MIQE guidelines for qPCR research reporting [68], as detailed in Supplementary Table 4.

### Genome-wide miRNA expression profiling

We performed miRNA expression profiling starting from 100 ng of total RNA. MiRNAs were labeled according to Microarray System protocol v.2.4 (Agilent Technologies). Briefly, Labeling Spike-In were added to 100 ng of total RNA and then dephosphorylated and labeled using microRNA Complete Labeling and Hyb Kit. Samples were then purified using Micro Bio-Spin 6 (Bio-Rad). After adding Hyb Spike-In, each sample was hybridized at 55°C for 20 hours on Human microRNA Microarray G4471A-029297 (Agilent Technologies), containing probes for 866 human miRNAs. After washing, slides were scanned by Agilent G2565CA scanner and images processed by Feature Extraction (FE) software using Agilent HD_miRNA protocol (Agilent Technologies). Raw and normalized data were deposited at National Center for Biotechnology Information Gene Expression Omnibus (GEO, http://www.ncbi.nlm.nih.gov/geo/) and are accessible through GEO accession number GSE86889, containing GSE84841 and GSE86652 that refer to NB cell lines and NB samples, respectively.

### Statistical analysis of miRNA expression

Tab-delimited text files containing FE results were acquired and analyzed in R v3.1.3 software environment (www.r-project.org) using the *limma* package [69] of Bioconductor (www.bioconductor.org). NB cell lines analysis included 16 text files corresponding to the two groups identified on the basis of the different ALK expression. ALK+ subgroup included the following cell lines: GILIN, KELLY, IMR5, IMR32, LAN1, LAN5, UKF-NB3, SH-SY5Y, SKNBE2, SKNSH, whereas ALK- subgroup included the following: ACN, GICAN, GIMEN, LA1-5S, SKNAS, SKNBE2C. Tumor samples analysis included 22 text files corresponding to the two groups identified on the basis of the different ALK expression. ALK+ group comprised the following samples: 2716, 2841, 2968, 2653, 2230, 2240, 2854, 2200, 2271, 2368, 2212, whereas ALK- group comprised the following samples: 2996, 2916, 2624, 2616, 2265, 2731, 2347, 2221, 2503, 2674, 2243. From such tab-delimited text files, only probes whose name starts with ‘hsa’ (FE field name ProbeName) were considered for further analysis (14192 features). To perform a quantitative analysis, only spots that satisfied the quality constraints showed in Supplementary Table 3 were used in the subsequent analysis as ‘detected’ spot. Background was subtracted from signal for each detected spot (we used the value with FE field name gBGSubSignal). Probes with less than 5 of detected spots across all arrays in one of the two groups (ALK+ and ALK-) were removed from the analysis. In NB cell lines 11701 probes were removed and 2491 were kept for further analysis, whereas in NB tumor samples 6992 probes were removed and 7200 were kept. Samples with a number of detected spots smaller than the MEAN-2SD of all the spots distribution across arrays were removed (SKNBE2 cell line and samples 2731 and 2347 were removed). Background corrected intensities of duplicate spots on each array were averaged (total number of averaged probes was 388 in NB cell lines and 675 in tumor samples). Data were then log2-transformed and normalized for between-array comparison using quantile normalization [70] and scale normalization [71, 72] for NB cell lines and tumor samples, respectively. Differential expression analysis between ALK+ and ALK- categories was carried out using Student’s t-test (unpaired, two-tailed, unequal variance). MiRNAs with raw p-values < 0.05 were selected: 71 miRNAs in NB cell lines and 43 miRNAs in tumor samples. We used hierarchical clustering to detect similarity relationships in microRNA log2-transformed expressions. Agglomerative hierarchical clusters were computed using the Euclidean distance between single vectors and the Ward method [73].

### MiRNA qPCR

We carried out qPCR to validate microarray results. cDNA was retro-transcribed from NB cell lines and tumor samples and amplified with a miRNA-specific miScript Primer Assay and the miScript SYBR Green PCR Kit (Qiagen), containing the miScript Universal Primer and QuantiTect SYBR Green PCR Master Mix, in a final volume of 20 μl. The miRNA mature-specific miScript Primers were specific for the following miRNAs: let-7g-5p, let7i-5p, miR-21-5p, miR-22-3p, miR-23a-3p, miR-29a-3p, miR-29b-3p, miR-29b-1-5p, miR-100-5p, miR-221-3p, miR-424-5p, miR-451a, miR-503-5p, miR-520e and miR-526b-3p. For miScript PCR Controls we used SNORD95 snoRNA and the RNU6 snRNA. The comparative ΔCt method was then applied.

### Cloning of ALK 3′-UTR

To generate the pMIR-ALK-UTR vector, the full-length wild type sequence of *ALK*-3′-UTR was amplified by PCR from genomic DNA of UKF-NB3 cell line. Forward primer (5′-AATTACTAGTgctcggtcgcacactcacttc-3′) was designed with a SpeI restriction site and reverse primer (5′-AATTAAGCTTTTagtcattacaaataactcc-3′) with a HindIII restriction site. Both restriction enzymes SpeI and HindIII were from New England Biolabs. *ALK* 3′-UTR was then cloned into the 3′-UTR polylinker site of the Firefly luciferase expressing vector pMIR-REPORT™ Luciferase (Life Technologies). Sanger sequencing was performed on the pMIR-REPORT-*ALK*-3′-UTR vector to verify that no mutation was introduced.

### Dual luciferase assay

Luciferase assay was carried out in SKNAS cell line that showed no endogenous expression of ALK. Cells were seeded in 96-Well Optical-Bottom Plates with Polymer Base suitable for luminometer assay (Thermo Scientific). Transient transfections of either the pMIR-REPORT-ALK-3′-UTR or the empty pMIR-REPORT along with a pGL4.75 vector containing the renilla luciferase, employed for normalization of results, were performed 24 hours after cell seeding. Cells were co-transfected with miR-424-5p or miR-503-5p double stranded synthetic miRNA mimics (Qiagen) using Attractene transfection reagent (Qiagen), according to the manufacturer’s protocol. Cells transfected with AllStars Negative Control siRNA (Qiagen) and untransfected cells were used as negative controls. Firefly and renilla luciferase activities were assessed 48 and 72 hours post transfection using the Dual Luciferase Reporter Assay System (Promega). For the luciferase assay, 20 μl of PLB buffer 1X (Passive Lysis Buffer, Promega) were added to each well and plates incubated for 15 minutes at room temperature on a thermomixer with the shaker on. Then, 20 μl of Firefly luciferase substrate (Luciferase Assay Reagent, LARII, Promega) were added to each well and light units measured by the plate reader Centro XS^3^ LB 960 Microplate Luminometer (Berthold Technologies). After firefly luciferase reading, 20 μl of the renilla luciferase substrate (Stop&Glow buffer, Promega) were added and light intensities measured. Each miRNA transfection was performed in 6 to 12 replicates and repeated in three independent experiments. Firefly luciferase was normalized to renilla luciferase activity, and then normalized to the luciferase activity ratio observed in the empty vector. Negative Control siRNA values were set as 100%.

### miRNA mimics transfection

SKNSH, NB1 and SKNAS cell lines, showing either detectable or high levels of ALK expression, or no ALK expression, respectively, were transfected with miR-424-5p and miR-503-5p mimics (Qiagen), in parallel with a negative control (AllStars Negative Control siRNA, Qiagen), using HiPerFect transfection reagent (Qiagen), according to the manufacturer’s protocol. Transfections were performed in T25 flasks with 1.5×10^6^ cells. Complexes composed by miRNA mimics and HiPerFect reagent were separately prepared, incubated at room temperature for 15 minutes and added directly to cell medium after cell seeding at a final concentration of 20 nM.

### Western blotting

A standard western blot protocol was carried out to evaluate ALK protein expression in cell lysates of NB cell lines. After 48 and 72 hours from transfection, cells were washed twice with ice-cold PBS and lysed with 50-100 μl of lysis buffer (Invitrogen), supplemented with a cocktail of protease inhibitors and PMSF (phenylmethylsulfonyl fluoride) (Sigma-Aldrich). Lysates were vortexed every 10 minutes and incubated on ice, for a total of 30 minutes, and then cleared by spinning. Proteins were separated in Mini-PROTEAN® TGX™ Precast 4-20% Gels (Biorad), and transfer was performed by Trans-Blot Turbo transfer system (Biorad). Membranes were incubated with primary antibodies for ALK (Cell Signaling) and GAPDH (Cell Signaling), which was used as a control for equal protein loading. Goat anti-rabbit IgG HRP conjugated secondary antibody was from Santa Cruz Biotechnologies. Blots were developed with Amersham’s ECL Prime (GE Healthcare) and images acquired by a chemiluminescent detection system (Uvitec Cambridge). Quantification of western blot bands was performed by ImageJ [74].

### MTT assay

Cells were seeded at a concentration ranging from 1×10^4^ to 1.5×10^4^ per well in six replicates, transfected in 96-well plates and additioned with 20 μl of 5 mg/l MTT at 48 and 72 hours post-transfection and incubated for 4 hours. Then, 200 μl of DMSO were added to each well and absorbance was read at 595 nm and background at 655 nm. Background values were then subtracted from MTT readings and each sample was compared to the negative control.

### Cell cycle analysis

Human NB cell lines (10^6^) were harvested 48 hours after transfection, washed twice with cold PBS, fixed in 70% ethyl alcohol and kept at 4°C. Cells were then washed twice in cold PBS and incubated 30 minutes at room temperature, in the dark, in a PBS based solution containing 0.05 mg/ml propidium iodide, 0.05% Triton X100 and 0.2 mg/ml RNAse A. After incubation, cells were washed in PBS and prepared for the cytometric analysis. Cell cycles were run on a FACScan and DNA content was analyzed using ModeFIT.

## SUPPLEMENTARY FIGURES AND TABLES





## Abbreviations

18S: RNA, 18S ribosomal
6FAM: 6-Carboxyfluorescein
ABL: ABL proto-oncogene 1, non-receptor tyrosine kinase
AKT: AKT serine/threonine kinase
ALCL: Anaplastic large cell lymphoma
ALK: Anaplastic lymphoma receptor tyrosine kinase
ATP: Adenosine triphosphate
BCR: BCR, RhoGEF and GTPase activating protein
Ct: Cycle Threshold
DMSO: Dimethyl sulfoxide
ECL: Electrochemiluminescence
FBS: Fetal bovine serum
FDA: Food and drug administration
FE: Feature extraction
GAPDH: Glyceraldehyde-3-phosphate dehydrogenase
GEO: Gene expression omnibus
IgG HRP: Horseradish peroxidase IgG conjugated
miRNA: microRNA
mTOR: Mechanistic target of rapamycin
MTT: 3-(4,5-dimethylthiazol-2-yl)-2,5-diphenyltetrazolium bromide
MYCN: v-myc avian myelocytomatosis viral oncogene neuroblastoma derived homolog
NB: Neuroblastoma
NPM: Nucleophosmin
PI3K: Phosphatidylinositol-4,5-bisphosphate 3-kinase
PLB: Passive lysis buffer
PMSF: Phenylmethylsulfonyl fluoride
qPCR: Quantitative polymerase chain reaction
RIN: RNA integrity number
RT: Reverse transcription
RTK: Receptor tyrosine kinase
SD: Standard deviation
siRNA: Small interfering RNA
snoRNA: Small nucleolar RNA
snRNA: Small nuclear RNA
UBC: Ubiquitin C
UTR: Untranslated region.

## References

[1] Bosse KR, Maris JM (2016). Advances in the translational genomics of neuroblastoma: From improving risk stratification and revealing novel biology to identifying actionable genomic alterations. Cancer.

[2] Maris JM, Hogarty MD, Bagatell R, Cohn SL (2007). Neuroblastoma. Lancet.

[3] Brodeur GM, Seeger RC, Schwab M, Varmus HE, Bishop JM (1984). Amplification of N-myc in untreated human neuroblastomas correlates with advanced disease stage. Science.

[4] Seeger RC, Brodeur GM, Sather H, Dalton A, Siegel SE, Wong KY, Hammond D (1985). Association of multiple copies of the N-myc oncogene with rapid progression of neuroblastomas. N Engl J Med.

[5] Huang M, Weiss WA (2013). Neuroblastoma and MYCN. Cold Spring Harb Perspect Med.

[6] Webb TR, Slavish J, George RE, Look AT, Xue L, Jiang Q, Cui X, Rentrop WB, Morris SW (2009). Anaplastic lymphoma kinase: role in cancer pathogenesis and small-molecule inhibitor development for therapy. Expert Rev Anticancer Ther.

[7] Morris SW, Kirstein MN, Valentine MB, Dittmer KG, Shapiro DN, Saltman DL, Look AT (1994). Fusion of a kinase gene, ALK, to a nucleolar protein gene, NPM, in non-Hodgkin’s lymphoma. Science.

[8] Griffin CA, Hawkins AL, Dvorak C, Henkle C, Ellingham T, Perlman EJ (1999). Recurrent involvement of 2p23 in inflammatory myofibroblastic tumors. Cancer Res.

[9] Soda M, Choi YL, Enomoto M, Takada S, Yamashita Y, Ishikawa S, Fujiwara S, Watanabe H, Kurashina K, Hatanaka H, Bando M, Ohno S, Ishikawa Y (2007). Identification of the transforming EML4-ALK fusion gene in non-small-cell lung cancer. Nature.

[10] Laurent C, Do C, Gascoyne RD, Lamant L, Ysebaert L, Laurent G, Delsol G, Brousset P (2009). Anaplastic lymphoma kinase-positive diffuse large B-cell lymphoma: a rare clinicopathologic entity with poor prognosis. J Clin Oncol.

[11] Mossé YP, Laudenslager M, Longo L, Cole KA, Wood A, Attiyeh EF, Laquaglia MJ, Sennett R, Lynch JE, Perri P, Laureys G, Speleman F, Kim C (2008). Identification of ALK as a major familial neuroblastoma predisposition gene. Nature.

[12] Janoueix-Lerosey I, Lequin D, Brugières L, Ribeiro A, de Pontual L, Combaret V, Raynal V, Puisieux A, Schleiermacher G, Pierron G, Valteau-Couanet D, Frebourg T, Michon J (2008). Somatic and germline activating mutations of the ALK kinase receptor in neuroblastoma. Nature.

[13] George RE, Sanda T, Hanna M, Fröhling S, Luther W, Zhang J, Ahn Y, Zhou W, London WB, McGrady P, Xue L, Zozulya S, Gregor VE (2008). Activating mutations in ALK provide a therapeutic target in neuroblastoma. Nature.

[14] Chen Y, Takita J, Choi YL, Kato M, Ohira M, Sanada M, Wang L, Soda M, Kikuchi A, Igarashi T, Nakagawara A, Hayashi Y, Mano H, Ogawa S (2008). Oncogenic mutations of ALK kinase in neuroblastoma. Nature.

[15] Schleiermacher G, Javanmardi N, Bernard V, Leroy Q, Cappo J, Rio Frio T, Pierron G, Lapouble E, Combaret V, Speleman F, de Wilde B, Djos A, Ora I (2014). Emergence of new ALK mutations at relapse of neuroblastoma. J Clin Oncol.

[16] Schramm A, Koster J, Assenov Y, Althoff K, Peifer M, Mahlow E, Odersky A, Beisser D, Ernst C, Henssen AG, Stephan H, Schroder C, Heukamp L (2015). Mutational dynamics between primary and relapse neuroblastomas. Nat Genet.

[17] Mossé YP (2016). Anaplastic Lymphoma Kinase as a Cancer Target in Pediatric Malignancies. Clin Cancer Res.

[18] Bresler SC, Weiser DA, Huwe PJ, Park JH, Krytska K, Ryles H, Laudenslager M, Rappaport EF, Wood AC, McGrady PW, Hogarty MD, London WB, Radhakrishnan R (2014). ALK mutations confer differential oncogenic activation and sensitivity to ALK inhibition therapy in neuroblastoma. Cancer Cell.

[19] Kwak EL, Bang YJ, Camidge DR, Shaw AT, Solomon B, Maki RG, Ou SH, Dezube BJ, Jänne PA, Costa DB, Varella-Garcia M, Kim WH, Lynch TJ (2010). Anaplastic lymphoma kinase inhibition in non-small-cell lung cancer. N Engl J Med.

[20] Mosse YP, Lim MS, Voss SD, Wilner K, Ruffner K, Laliberte J, Rolland D, Balis FM, Maris JM, Weigel BJ, Ingle AM, Ahern C, Adamson PC (2013). Safety and activity of crizotinib for paediatric patients with refractory solid tumours or anaplastic large-cell lymphoma: a Children’s Oncology Group phase 1 consortium study. Lancet Oncol.

[21] Fontana D, Ceccon M, Gambacorti-Passerini C, Mologni L (2015). Activity of second-generation ALK inhibitors against crizotinib-resistant mutants in an NPM-ALK model compared to EML4-ALK. Cancer Med.

[22] Lamant L, Pulford K, Bischof D, Morris SW, Mason DY, Delsol G, Mariamé B (2000). Expression of the ALK tyrosine kinase gene in neuroblastoma. Am J Pathol.

[23] Passoni L, Longo L, Collini P, Coluccia AM, Bozzi F, Podda M, Gregorio A, Gambini C, Garaventa A, Pistoia V, Del Grosso F, Tonini GP, Cheng M (2009). Mutation-independent anaplastic lymphoma kinase overexpression in poor prognosis neuroblastoma patients. Cancer Res.

[24] Schulte JH, Bachmann HS, Brockmeyer B, De Preter K, Oberthur A, Ackermann S, Kahlert Y, Pajtler K, Theissen J, Westermann F, Vandesompele J, Speleman F, Berthold F (2011). High ALK receptor tyrosine kinase expression supersedes ALK mutation as a determining factor of an unfavorable phenotype in primary neuroblastoma. Clin Cancer Res.

[25] Montavon G, Jauquier N, Coulon A, Peuchmaur M, Flahaut M, Bourloud KB, Yan P, Delattre O, Sommer L, Joseph JM, Janoueix-Lerosey I, Gross N, Mühlethaler-Mottet A (2014). Wild-type ALK and activating ALK-R1275Q and ALK-F1174L mutations upregulate Myc and initiate tumor formation in murine neural crest progenitor cells. Oncotarget.

[26] Alvarez-Garcia I, Miska EA (2005). MicroRNA functions in animal development and human disease. Development.

[27] Zhang B, Pan X, Cobb GP, Anderson TA (2007). microRNAs as oncogenes and tumor suppressors. Dev Biol.

[28] Calin GA, Dumitru CD, Shimizu M, Bichi R, Zupo S, Noch E, Aldler H, Rattan S, Keating M, Rai K, Rassenti L, Kipps T, Negrini M (2002). Frequent deletions and down-regulation of micro- RNA genes miR15 and miR16 at 13q14 in chronic lymphocytic leukemia. Proc Natl Acad Sci U S A.

[29] Calin GA, Croce CM (2006). MicroRNA signatures in human cancers. Nat Rev Cancer.

[30] Stallings RL, Foley NH, Bryan K, Buckley PG, Bray I (2010). Therapeutic targeting of miRNAs in neuroblastoma. Expert Opin Ther Targets.

[31] Schulte JH, Horn S, Schlierf S, Schramm A, Heukamp LC, Christiansen H, Buettner R, Berwanger B, Eggert A (2009). MicroRNAs in the pathogenesis of neuroblastoma. Cancer Lett.

[32] Powers JT, Tsanov KM, Pearson DS, Roels F, Spina CS, Ebright R, Seligson M, de Soysa Y, Cahan P, TheiΔen J, Tu HC, Han A, Kurek KC (2016). Multiple mechanisms disrupt the let-7 microRNA family in neuroblastoma. Nature.

[33] Soriano A, París-Coderch L, Jubierre L, Martínez A, Zhou X, Piskareva O, Bray I, Vidal I, Almazán-Moga A, Molist C, Roma J, Bayascas JR, Casanovas O (2016). MicroRNA-497 impairs the growth of chemoresistant neuroblastoma cells by targeting cell cycle, survival and vascular permeability genes. Oncotarget.

[34] Naraparaju K, Kolla V, Zhuang T, Higashi M, Iyer R, Kolla S, Okawa ER, Blobel GA, Brodeur GM (2016). Role of microRNAs in epigenetic silencing of the CHD5 tumor suppressor gene in neuroblastomas. Oncotarget.

[35] Bresler SC, Wood AC, Haglund EA, Courtright J, Belcastro LT, Plegaria JS, Cole K, Toporovskaya Y, Zhao H, Carpenter EL, Christensen JG, Maris JM, Lemmon MA (2011). Differential inhibitor sensitivity of anaplastic lymphoma kinase variants found in neuroblastoma. Sci Transl Med.

[36] Moore NF, Azarova AM, Bhatnagar N, Ross KN, Drake LE, Frumm S, Liu QS, Christie AL, Sanda T, Chesler L, Kung AL, Gray NS, Stegmaier K, George RE (2014). Molecular rationale for the use of PI3K/AKT/mTOR pathway inhibitors in combination with crizotinib in ALK-mutated neuroblastoma. Oncotarget.

[37] Siaw JT, Wan H, Pfeifer K, Rivera VM, Guan J, Palmer RH, Hallberg B (2016). Brigatinib, an anaplastic lymphoma kinase inhibitor, abrogates activity and growth in ALK-positive neuroblastoma cells, Drosophila and mice. Oncotarget.

[38] Aveic S, Pantile M, Seydel A, Esposito MR, Zanon C, Li G, Tonini GP (2016). Combating autophagy is a strategy to increase cytotoxic effects of novel ALK inhibitor entrectinib in neuroblastoma cells. Oncotarget.

[39] Iyer R, Wehrmann L, Golden RL, Naraparaju K, Croucher JL, MacFarland SP, Guan P, Kolla V, Wei G, Cam N, Li G, Hornby Z, Brodeur GM (2016). Entrectinib is a potent inhibitor of Trk-driven neuroblastomas in a xenograft mouse model. Cancer Lett.

[40] Di Paolo D, Yang D, Pastorino F, Emionite L, Cilli M, Daga A, Destafanis E, Di Fiore A, Piaggio F, Brignole C, Xu X, Liang C, Gibbons J (2015). New therapeutic strategies in neuroblastoma: combined targeting of a novel tyrosine kinase inhibitor and liposomal siRNAs against ALK. Oncotarget.

[41] Forrest AR, Kanamori-Katayama M, Tomaru Y, Lassmann T, Ninomiya N, Takahashi Y, de Hoon MJL, Kubosaki A, Kaiho A, Suzuki M, Yasuda J, Kawai J, Hayashizaki Y (2010). Induction of microRNAs, mir-155, mir-222, mir-424 and mir-503, promotes monocytic differentiation through combinatorial regulation. Leukemia.

[42] Finnerty JR, Wang WX, Hébert SS, Wilfred BR, Mao G, Nelson PT (2010). The miR-15/107 group of microRNA genes: evolutionary biology, cellular functions, and roles in human diseases. J Mol Biol.

[43] Gupta A, Hossain MM, Read DE, Hetz C, Samali A, Gupta S (2015). PERK regulated miR-424(322)-503 cluster fine-tunes activation of IRE1 and ATF6 during Unfolded Protein Response. Sci Rep.

[44] Rosa A, Ballarino M, Sorrentino A, Sthandier O, De Angelis FG, Marchioni M, Masella B, Guarini A, Fatica A, Peschle C, Bozzoni I (2007). The interplay between the master transcription factor PU.1 and miR-424 regulates human monocyte/macrophage differentiation. Proc Natl Acad Sci USA.

[45] Sarkar S, Dey BK, Dutta A (2010). MiR-322/424 and -503 are induced during muscle differentiation and promote cell cycle quiescence and differentiation by down-regulation of Cdc25A. Mol Biol Cell.

[46] Ghosh G, Subramanian IV, Adhikari N, Zhang X, Joshi HP, Basi D, Chandrashekhar YS, Hall JL, Roy S, Zeng Y, Ramakrishnan S (2010). Hypoxia-induced microRNA-424 expression in human endothelial cells regulates HIF-α isoforms and promotes angiogenesis. J Clin Invest.

[47] Liu Q, Fu H, Sun F, Zhang H, Tie Y, Zhu J, Xing R, Sun Z, Zheng X (2008). miR-16 family induces cell cycle arrest by regulating multiple cell cycle genes. Nucleic Acids Res.

[48] Pallasch CP, Patz M, Park YJ, Hagist S, Eggle D, Claus R, Debey-Pascher S, Schulz A, Frenzel LP, Claasen J, Kutsch N, Krause G, Mayr C (2009). miRNA deregulation by epigenetic silencing disrupts suppression of the oncogene PLAG1 in chronic lymphocytic leukemia. Blood.

[49] Imig J, Motsch N, Zhu JY, Barth S, Okoniewski M, Reineke T, Tinguely M, Faggioni A, Trivedi P, Meister G, Renner C, Grässer FA (2011). microRNA profiling in Epstein-Barr virus-associated B-cell lymphoma. Nucleic Acids Res.

[50] Xu J, Li Y, Wang F, Wang X, Cheng B, Ye F, Xie X, Zhou C, Lu W (2013). Suppressed miR-424 expression via upregulation of target gene Chk1 contributes to the progression of cervical cancer. Oncogene.

[51] Ruiz-Llorente L, Ardila-González S, Fanjul LF, Martínez-Iglesias O, Aranda A (2014). microRNAs 424 and 503 are mediators of the anti-proliferative and anti-invasive action of the thyroid hormone receptor beta. Oncotarget.

[52] Zhou J, Wang W (2011). Analysis of microRNA expression profiling identifies microRNA-503 regulates metastatic function in hepatocellular cancer cell. J Surg Oncol.

[53] Yu L, Ding GF, He C, Sun L, Jiang Y, Zhu L (2014). MicroRNA-424 is down-regulated in hepatocellular carcinoma and suppresses cell migration and invasion through c-Myb. PLoS One.

[54] Cheung TH, Man KN, Yu MY, Yim SF, Siu NS, Lo KW, Doran G, Wong RR, Wang VW, Smith DI, Worley MJ, Berkowitz RS, Chung TK, Wong YF (2012). Dysregulated microRNAs in the pathogenesis and progression of cervical neoplasm. Cell Cycle.

[55] Faraoni I, Laterza S, Ardiri D, Ciardi C, Fazi F, Lo-Coco F (2012). MiR-424 and miR-155 deregulated expression in cytogenetically normal acute myeloid leukaemia: correlation with NPM1 and FLT3 mutation status. J Hematol Oncol.

[56] Xu S, Tao Z, Hai B, Liang H, Shi Y, Wang T, Song W, Chen Y, OuYang J, Chen J, Kong F, Dong Y, Jiang SW (2016). miR-424(322) reverses chemoresistance via T-cell immune response activation by blocking the PD-L1 immune checkpoint. Nat Commun.

[57] Oneyama C, Kito Y, Asai R, Ikeda J, Yoshida T, Okuzaki D, Kokuda R, Kakumoto K, Takayama K, Inoue S, Morii E, Okada M (2013). MiR-424/503-mediated Rictor upregulation promotes tumor progression. PLoS One.

[58] Berghmans T, Ameye L, Willems L, Paesmans M, Mascaux C, Lafitte JJ, Meert AP, Scherpereel A, Cortot AB, Cstoth I, Dernies T, Toussaint L, Leclercq N, Sculier JP (2013). European Lung Cancer Working Party. Identification of microRNA-based signatures for response and survival for non-small cell lung cancer treated with cisplatin-vinorelbine A ELCWP prospective study. Lung Cancer.

[59] Chen B, Duan L, Yin G, Tan J, Jiang X (2013). Simultaneously expressed miR-424 and miR-381 synergistically suppress the proliferation and survival of renal cancer cells---Cdc2 activity is up-regulated by targeting WEE1. Clinics (Sao Paulo).

[60] Long XH, Mao JH, Peng AF, Zhou Y, Huang SH, Liu ZL (2013). Tumor suppressive microRNA-424 inhibits osteosarcoma cell migration and invasion via targeting fatty acid synthase. Exp Ther Med.

[61] Wu CT, Lin WY, Chang YH, Lin PY, Chen WC, Chen MF (2015). DNMT1-dependent suppression of microRNA424 regulates tumor progression in human bladder cancer. Oncotarget.

[62] Wu K, Hu G, He X, Zhou P, Li J, He B, Sun W (2013). MicroRNA-424-5p suppresses the expression of SOCS6 in pancreatic cancer. Pathol Oncol Res.

[63] Hershkovitz-Rokah O, Modai S, Pasmanik-Chor M, Toren A, Shomron N, Raanani P, Shpilberg O, Granot G (2015). Restoration of miR-424 suppresses BCR-ABL activity and sensitizes CML cells to imatinib treatment. Cancer Lett.

[64] Zhang Y, Li T, Guo P, Kang J, Wei Q, Jia X, Zhao W, Huai W, Qiu Y, Sun L, Han L (2014). MiR-424-5p reversed epithelial-mesenchymal transition of anchorage-independent HCC cells by directly targeting ICAT and suppressed HCC progression. Sci Rep.

[65] Steinhilber J, Bonin M, Walter M, Fend F, Bonzheim I, Quintanilla-Martinez L (2015). Next-generation sequencing identifies deregulation of microRNAs involved in both innate and adaptive immune response in ALK+ ALCL. PLoS One.

[66] Vishwamitra D, Li Y, Wilson D, Manshouri R, Curry CV, Shi B, Tang XM, Sheehan AM, Wistuba II, Shi P, Amin HM (2012). MicroRNA 96 is a post-transcriptional suppressor of anaplastic lymphoma kinase expression. Am J Pathol.

[67] Croce CM (2009). Causes and consequences of microRNA dysregulation in cancer. Nat Rev Genet.

[68] Bustin SA, Benes V, Garson JA, Hellemans J, Huggett J, Kubista M, Mueller R, Nolan T, Pfaffl MW, Shipley GL, Vandesompele J, Wittwer CT (2009). The MIQE guidelines: Minimum Information for publication of Quantitative Real-Time PCR Experiments. Clin Chem.

[69] Smyth GK, Gentleman R, Carey V, Dudoit S, Irizarry R, Huber W (2005). Limma: linear models for microarray data. ‘Bioinformatics and Computational Biology Solutions using R and Bioconductor’.

[70] Bolstad BM, Irizarry RA, Astrand M, Speed TP (2003). A comparison of normalization methods for high density oligonucleotide array data based on variance and bias. Bioinformatics.

[71] Yang YH, Dudoit S, Luu P, Lin DM, Peng V, Ngai J, Speed TP (2002). Normalization for cDNA microarray data: a robust composite method addressing single and multiple slide systematic variation. Nucleic Acids Res.

[72] Yang YH, Thorne NP, Goldstein DR (2003). Normalization for two-color cDNA microarray data. Science and Statistics: A Festschrift for Terry Speed. IMS Lecture Notes: Monograph Series.

[73] Ward JH (1963). Hierachical grouping to optimize an objective function. J Am Stat Assoc.

[74] Abramoff MD, Magalhaes PJ, Ram SJ (2004). Image Processing with ImageJ. Biophotonics International.

