# Supplementary material for: A genome-wide microRNA profiling indicates miR-424-5p and miR-503-5p as regulators of ALK expression in neuroblastoma
Source: Oncotarget. 2017 Apr 11;8(34):56518–32. doi: 10.18632/oncotarget.17033 (PMC5593579; doi:10.18632/oncotarget.17033)
Supplement: Supplementary file 1 [file oncotarget-08-56518-s001.pdf]

A genome-wide microRNA profiling indicates miR-424-5p and miR-503-5p as regulators of ALK expression in neuroblastoma

SUPPLEMENTARY FIGURES AND TABLES

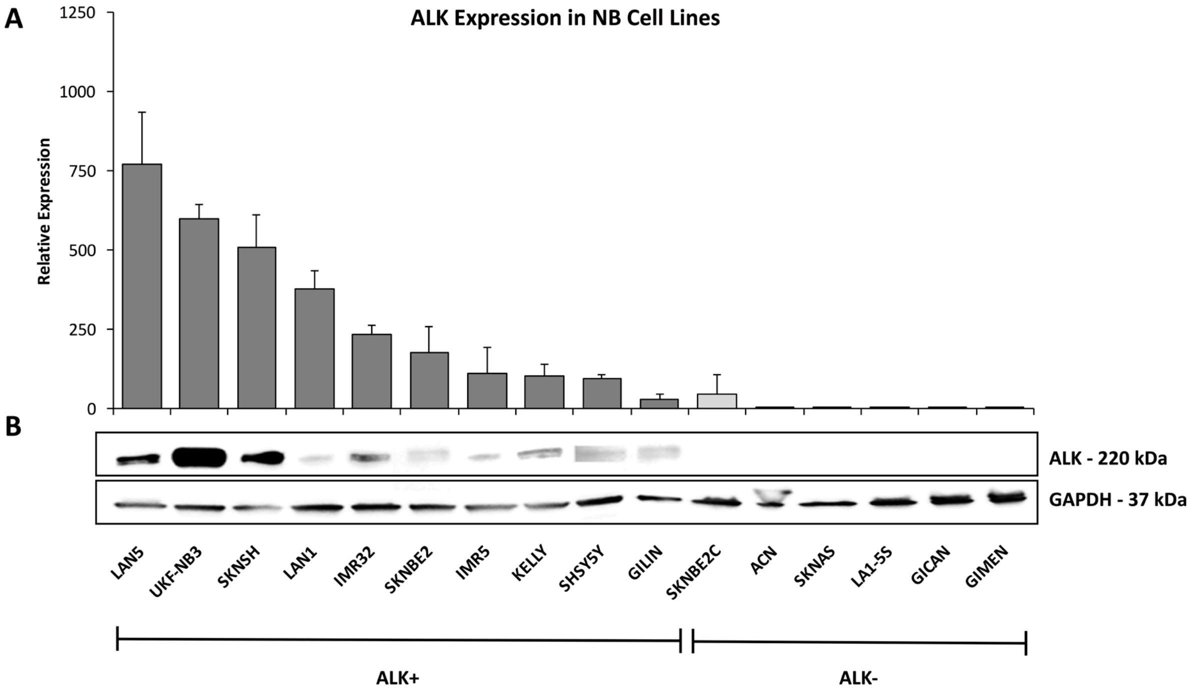

Supplementary Figure 1: Analysis of ALK expression and definition of ALK+ and ALK- subgroups in NB cell lines. *ALK* mRNA expression was performed by qPCR **(A)** and ALK protein detected by western blot **(B)**. Numbers in the Y-axis represents the fold change in expression with respect to the less expressed sample, which was set as 1.

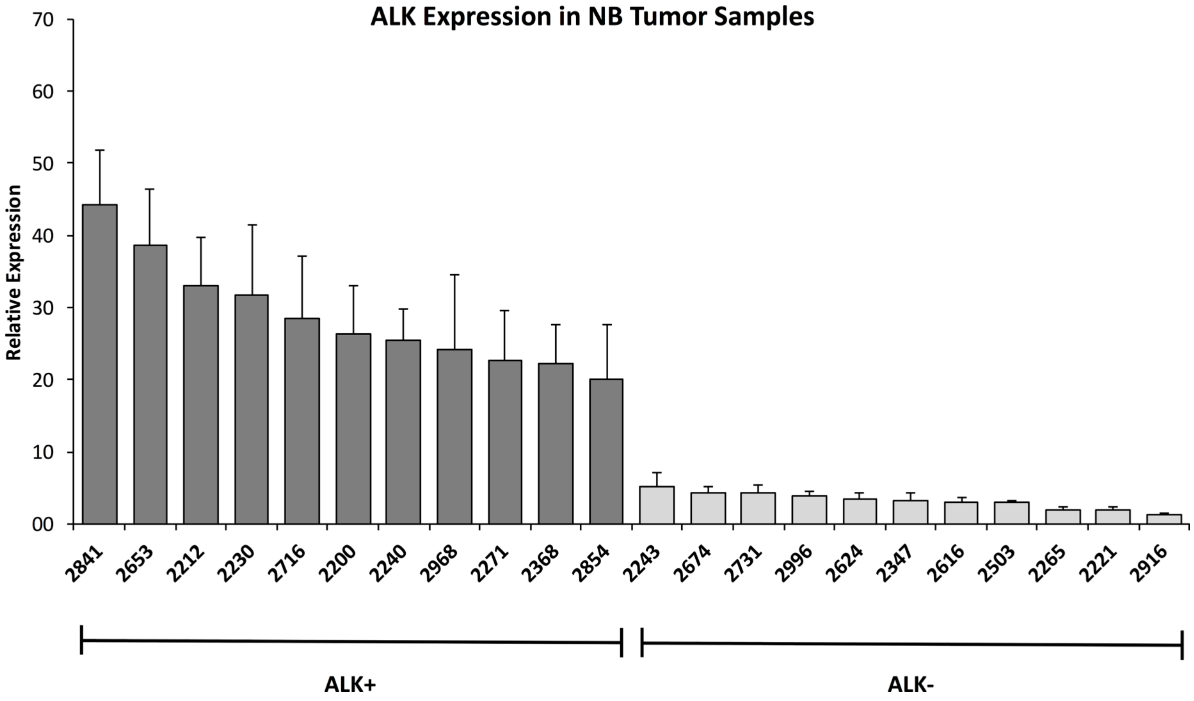

Supplementary Figure 2: Analysis of ALK expression and definition of ALK+ and ALK- subgroups in NB tumor samples. *ALK* mRNA expression was performed by qPCR. Numbers in the Y-axis represents the fold change in expression with respect to the less expressed sample, which was set as 1.

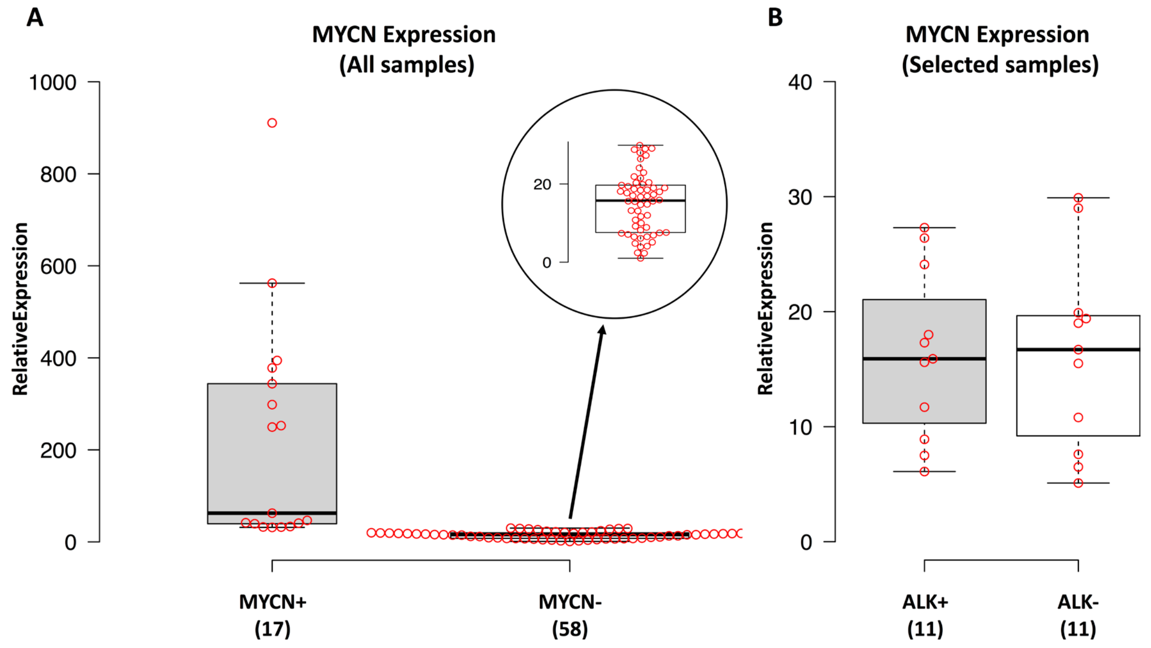

Supplementary Figure 3: *MYCN* expression in NB tumor samples. *MYCN* mRNA expression was performed by qPCR and data box-plotted using BoxPlotR. Numbers in the Y-axis represents the fold change in expression with respect to the less expressed sample, which was set as 1. **(A)** Seventeen samples that were either *MYCN* amplified or with high *MYCN* expression were discarded from miRNAs expression analysis. **(B)** Of the 58 samples with *MYCN* single copy and low *MYCN* expression, we selected 22 samples, with equal levels of *MYCN*, to determine the two groups of 11 samples showing a significant differential expression of *ALK* as shown in Figure 1 and Supplementary Figure 2.

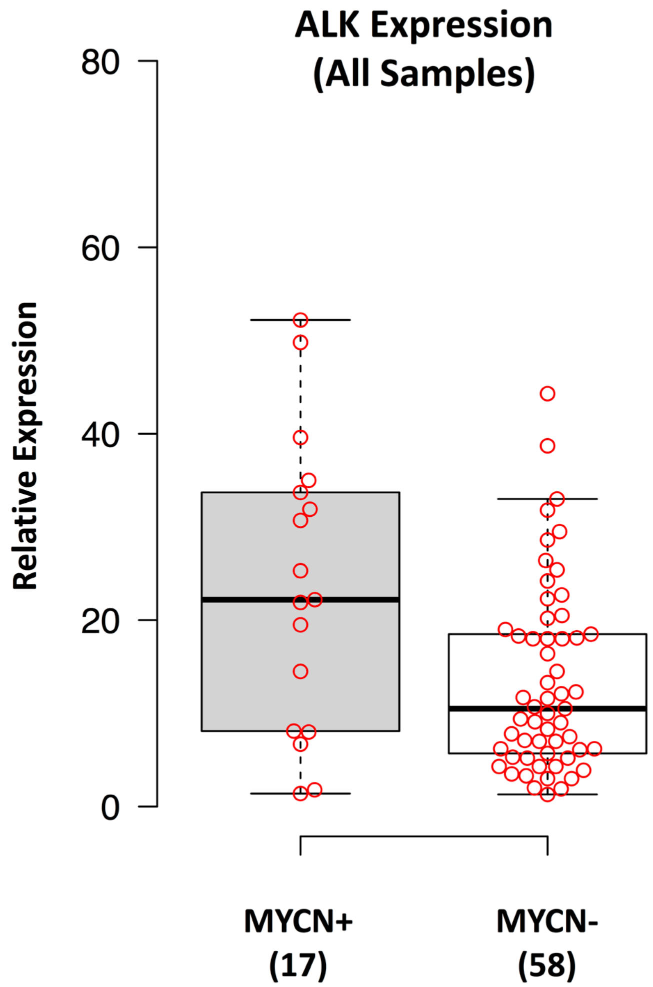

Supplementary Figure 4: ALK expression in NB tumor samples. *ALK* mRNA expression was performed by qPCR and data box-plotted using BoxPlotR. Numbers in the Y-axis represents the fold change in expression with respect to the less expressed sample, which was set as 1.

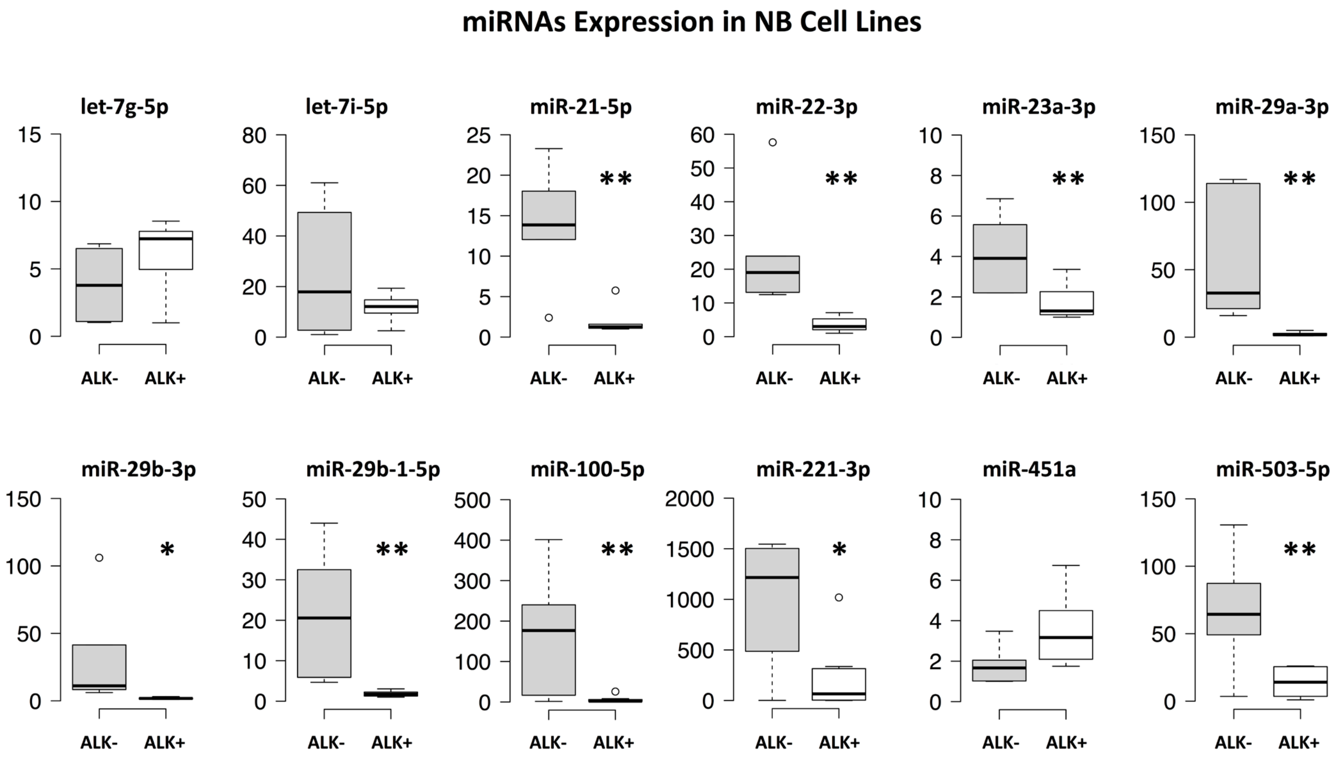

Supplementary Figure 5: Validation of candidate miRNAs in NB cell lines. Validation analysis of miRNAs expression was performed by qPCR and data were box-plotted using BoxPlotR. p-value < 0.05 (*); p-value < 0.01 (**).

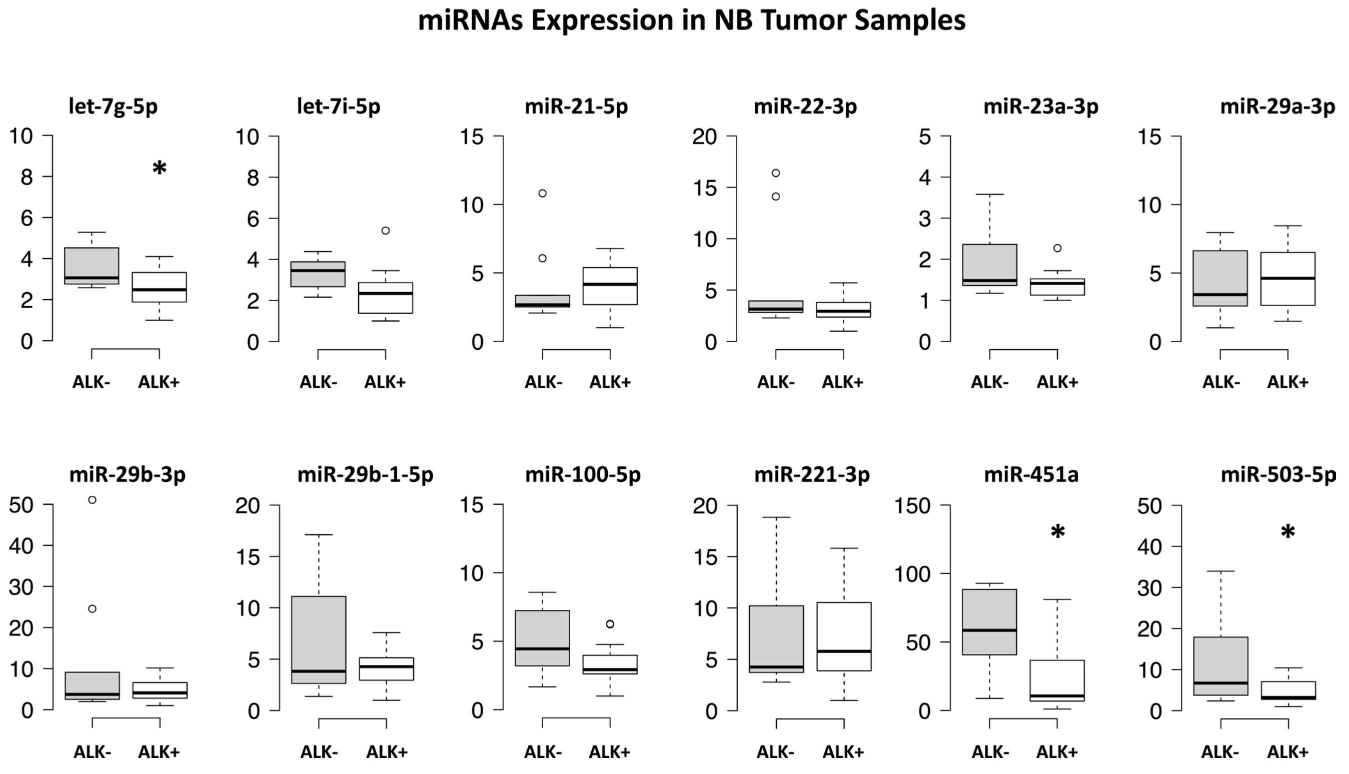

Supplementary Figure 6: Validation of candidate miRNAs in NB tumor samples. Validation analysis of miRNAs expression was performed by qPCR and data were box-plotted using BoxPlotR. p-value < 0.05 (*); p-value < 0.01 (**).

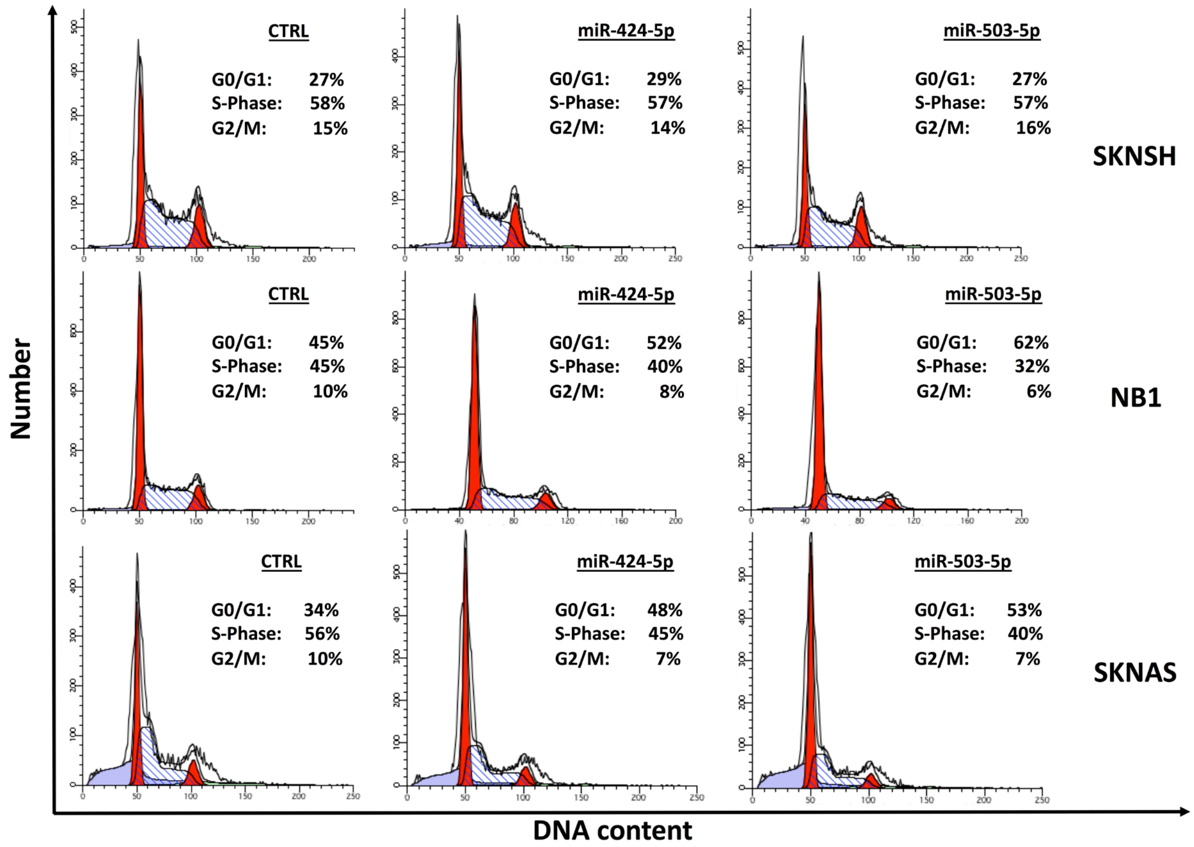

Supplementary Figure 7: Cell cycle DNA profile of ALK+ and ALK- NB cell lines. Cell cycle profiles were analyzed 48 hours after transfection. Untreated cells transfected with AllStars Negative Control siRNA (Qiagen), which has no homology to any known mammalian gene, were used as controls. The distribution and percentage of cells in G0/G1, S and G2/M phase of the cell cycle are indicated.

Supplementary Table 1: Cell lines information

Cell line	Origin	Tumor	MYCN Status
ACN	BBCF	NB	NA
GICAN	BBCF	NB	NA
GILIN	BBCF	NB	A
GIMEN	BBCF	NB	NA
IMR32	BBCF	NB	A
IMR5	BBCF	NB	A
KELLY	ECACC	NB	A
LA1-5S	ECACC	NB	A
LAN1	ECACC	NB	A
LAN5	BBCF	NB	A
NB1	R. Luksch Lab	NB	NA
SH-SY5Y	BBCF	NB	NA
SKNAS	BBCF	NB	NA
SKNBE2	BBCF	NB	A
SKNBE2(C)	BBCF	NB	A
SKNSH	BBCF	NB	NA
UKF-NB3	R. Luksch Lab	NB	A

BBCF: Biological Bank and Cell Factory, Core Facility of the IRCCS AOU San Martino-IST in Genoa (www.iclc.it); ECACC: European Collection of Cell Cultures (www.phe-culturecollections.org.uk/collections/ecacc.aspx).

A: Amplified; NA: Not Amplified.

Supplementary Table 2: NB samples information

NB sample ID	INSS Stage	Age at diagnosis (months)	Histology	MYCN Status	ALK mRNA expression
2200	4	165	NB	NA	High
2212	4	34	NB	NA	High
2221	1	2	NB	NA	Low
2230	4	49	GNB	NA	High
2240	3	10	NB	NA	High
2243	1	3	NB	NA	Low
2265	1	1	NB	NA	Low
2271	3	6	NB	NA	High
2347	3	4	NB	NA	Low
2368	4	75	NB	NA	High
2503	3	9	NB	NA	Low
2616	3	43	NB	NA	Low
2624	2	9	NB	NA	Low
2653	3	5	NB	NA	High
2674	4	39	NB	NA	Low
2716	3	2	NB	NA	High
2731	4	26	NB	NA	Low
2841	4	16	NB	NA	High
2854	4	51	NB	NA	High
2916	1	2	NB	NA	Low
2968	2	16	NB	NA	High
2996	2	17	NB	NA	Low

22 tumor samples were employed for miRNA screening and referred to the Gaslini Children's Hospital. All analyses were performed according to the Helsinki declaration. INSS: International Neuroblastoma Staging System); NB: Neuroblastoma; GNB: Ganglioneuroblastoma; NA: Not Amplified.

Supplementary Table 3: Quality control feature filtering used in the analysis

Criteria	Value	Criteria meaning
ControlType	=0	Spot is not a Control type
IsManualFlag	=0	Spot has not been flagged manually as a bad quality spot
gBGSubSignal	≥0	gBGSubSignal = gMeanSignal - gBGUsed) means that the Background-subtracted signal is larger or equal to zero
gIsFeatPopnOL	=0	Feature is not a Population Outlier Probes with replicate features on a microarray are examined using population statistics. A feature is a population outlier if its signal is less than a lower threshold or exceeds an upper threshold determined using a multiplier (1.42) times the interquartile range (i.e., IQR) of the population
gIsBGPopnOL	=0	Background of the feature is not a Population Outlier (same concept as above for the background)
gIsFeatNonUnifOL	=0	Feature is not a non-uniformity outlier in g(r). A feature is non-uniform if the pixel noise of feature exceeds a threshold established for a "uniform" feature
gIsBGNonUnifOL	=0	Local background is not a non-uniformity outlier in g(r) (same concept as above for the background)
gIsSaturated	=0	Feature is not saturated. A feature is saturated if 50% of the pixels in a feature are above the saturation threshold

Detected spots are those satisfying the criteria detailed in the table.

Supplementary Table 4: MIQE checklist

See Supplementary File 1
