# Supplementary material for: A genome-wide microRNA profiling indicates miR-424-5p and miR-503-5p as regulators of ALK expression in neuroblastoma
Source: Oncotarget. 2017 Apr 11;8(34):56518–32. doi: 10.18632/oncotarget.17033 (PMC5593579; doi:10.18632/oncotarget.17033)
Supplement: Supplementary file 2 [file oncotarget-08-56518-s002.docx]

| **Supplementary Table 4 – MIQE checklist** |  |  |
| --- | --- | --- |
|  |  |  |
| **Item to check** | **Importance** | **Checklist** |
| **EXPERIMENTAL DESIGN** |  |  |
| Definition of experimental and control groups | **E** | 1. Relative quantification of ALK gene in 16 NB cell lines 2. Relative quantification of ALK gene in 75 NB tumor samples 3. Relative quantification of miRNAs in 16 NB cell lines divided in ALK+ subgroup (GILIN, KELLY, IMR5, IMR32, LAN1, LAN5, UKF-NB3, SH-SY5Y, SKNBE2, SKNSH) and in ALK- subgroup (ACN, GICAN, GIMEN, LA1-5S, SKNAS, SKNBE2C) 4. Relative quantification of miRNAs in 22 NB patient samples divided in ALK+ subgroup (IDs: 2716, 2841, 2968, 2653, 2230, 2240, 2854, 2200, 2271, 2368, 2212) and ALK- subgroup (IDs: 2996, 2916, 2624, 2616, 2265, 2731, 2347, 2221, 2503, 2674, 2243) |
| Number within each group | **E** | 1. 16 samples 2. 75 samples 3. 16 samples, 10 cell lines ALK+ and 6 ALK- 4. 22 samples, 11 NB samples ALK+ and 11 ALK- |
| Assay carried out by core lab or investigator's lab? | D | Assays were carried out by investigator's lab |
| Acknowledgement of authors' contributions | D | Marilena De Mariano performed all assays |
| **SAMPLE** |  |  |
| Description | **E** | For NB cell lines see Supplementary Table 1. Tumor specimens were collected at the onset of disease from 120 patients, who were diagnosed with a primary NB between 2002 and 2008, and referred to the Gaslini Children’s Hospital (Genoa, Italy) |
| Volume/mass of sample processed | D | 1-2 x10^6^ cells for NB cell lines  Not determined for tumor samples |
| Microdissection or macrodissection | **E** | Tumor samples included only specimens with a neuroblastic cell content >70% |
| Processing procedure | **E** | Cell lines were cultured at 37°C and 5% CO_2_ and maintained in RPMI 1640, supplemented with L-glutamine, penicillin/streptomycin, and 10% FBS (Lonza). 1% Trypsin/PBS was used to release cells. Cell suspension was centrifuged at 1,000 x g for 5 minutes to pellet the cells |
| If frozen - how and how quickly? | **E** | The pellet of cells was immediately frozen in dry ice and then stored at -80°C |
| If fixed - with what, how quickly? | **E** | Not fixed |
| Sample storage conditions and duration (especially for FFPE samples) | **E** | Cells were maintained at -80°C until RNA extraction |
| **NUCLEIC ACID EXTRACTION** |  |  |
| Procedure and/or instrumentation | **E** | Total RNA containing miRNAs was extracted by miRNeasy Mini kit (Qiagen), including a DNase step, according to the manufacturer’s instructions |
| Name of kit and details of any modifications | **E** | miRNeasy Mini kit (Qiagen) |
| Source of additional reagents used | D | None |
| Details of DNase treatment | **E** | On column RNase-free DNase I treatment: after Buffer RW1, 80 µl of DNase solution (10 µl DNase I stock solution (1500 units in 550 µl RNase-free water) and 70 µl Buffer RDD) were added to the RNeasy spin column membrane and incubated at room temperature for 15 minutes |
| Contamination assessment (DNA or RNA) | **E** | Non retro-transcribed RNAs were amplified under the same conditions to verify gDNA contamination |
| Nucleic acid quantification | **E** | RNA quantification was assessed by using Nanodrop ND-1000 spectrophotometer and by Bioanalyzer |
| Instrument and method | **E** | Nanodrop ND-1000 spectrophotometer (Thermo Scientific) according to instrument protocol. 2100 Bioanalyzer (Agilent Technologies) according to kit protocols |
| Purity (A260/A280) | D | About 2 for all samples |
| Yield | D | For cell lines from 12 µg to 50 µg, for NB samples from 4.5 µg to 15 µg |
| RNA integrity method/instrument | **E** | The integrity of total RNA and miRNAs was checked by RNA 6000 Nano and Small RNA kits, respectively, on 2100 Bioanalyzer (Agilent Technologies) |
| RIN/RQI or Cq of 3' and 5' transcripts | **E** | Only RNAs with a RIN ≥7 were included in subsequent experiments |
| Electrophoresis traces | D | Not performed |
| Inhibition testing (Cq dilutions, spike or other) | **E** | Not performed |
| **REVERSE TRANSCRIPTION** |  |  |
| Complete reaction conditions | **E** | 1 µg of total RNA, containing miRNAs, was retro-transcribed using miScript Reverse Transcription Kit (Qiagen) and miScript HiFlex Buffer in a final volume of 20 µl. A poly(A) tag was added during the reaction and reverse transcription (RT) was performed in presence of both oligo-dT and random primers. The oligo-dT primers had a universal tag sequence on the 5' end that allowed amplification in the qPCR step |
| Amount of RNA and reaction volume | **E** | 1 µg of total RNA in 20 µl reaction volume |
| Priming oligonucleotide (if using GSP) and concentration | **E** | 10x Nucleics Mix (2 µl in 20 µl of reaction volume, following miScript II RT kit instruction) |
| Reverse transcriptase and concentration | **E** | miScript Reverse Transcriptase Mix (2 µl in 20 µl of reaction volume, following miScript II RT kit instruction) |
| Temperature and time | **E** | Template RNA was added to each tube containing reverse-transcription master mix, containing miScript HiFlex Buffer, 10x Nucleics Mix, miScript Reverse Transcriptase Mix. Incubation for 60 min at 37ºC. Incubation for 5 min at 95ºC to inactivate miScript Reverse Transcriptase Mix. |
| Manufacturer of reagents and catalogue number | D | Qiagen Cat No 218061 |
| Cqs with and without RT | D* | RNAs without reverse transcription was undetectable |
| Storage conditions of cDNA | D | -20°C |
| **qPCR TARGET INFORMATION** |  | See Ref 33 |
| If multiplex, efficiency and LOD of each assay. | **E** | Not applicable since we performed single-plex qPCRs |
| Sequence accession number | **E** | The miRNA mature-specific miScript Primers were specific for the following miRNAs: let-7g-5p, let7i-5p, miR-21-5p, miR-22-3p, miR-23a-3p, miR-29a-3p, miR-29b-3p, miR-29b-1-5p, miR-100-5p, miR-221-3p, miR-424-5p, miR-451a, miR-503-5p, miR-520e and miR-526b-3p. For miScript PCR controls we used SNORD95 snoRNA and the RNU6 snRNA. For ALK we used NM_004304.4, for house-keeping controls we used 18S, GAPDH, and UBC. |
| Location of amplicon | D |  |
| Amplicon length | **E** | ALK 125 bp |
| In silico specificity screen (BLAST, etc) | **E** | Yes |
| Pseudogenes, retropseudogenes or other homologs? | D |  |
| Sequence alignment | D |  |
| Secondary structure analysis of amplicon | D |  |
| Location of each primer by exon or intron (if applicable) | **E** | Exon Boundary 26 - 27 |
| What splice variants are targeted? | **E** |  |
| **qPCR OLIGONUCLEOTIDES** |  |  |
| Primer sequences | **E** | Human miScript Primer for: let-7g-5p (Cat No MS00008337), let7i-5p (Cat No MS00003157), miR-21-5p (Cat No MS00009079), miR-22-3p (Cat No MS00003220), miR-29a-3p (Cat No MS00003262), miR-29b-3p (Cat No MS00006566), miR-29b-1-5p (Cat No MS00009289), miR-100-5p (Cat No MS00031234), miR-221-3p (Cat No MS00003857), miR-424-5p (Cat No MS00004186), miR-451a (Cat No MS00004242), miR-503-5p (Cat No MS00033838), miR-520e (Cat No MS00004536), miR-526b-3p (Cat No MS00031976), SNORD95 snoRNA (Cat No MS00033726), RNU6 snRNA (Cat No MS00033740) and miScript Universal Primer (miScript PCR kit, Qiagen)  Human FAM TaqMan Assays specific for ALK (assay ID Hs00608291_m1, Applied Biosystems)  Human FAM Double-Dye Assays specific for 18S, GAPDH, and UBC (Primer Design) |
| RTPrimerDB Identification Number | D |  |
| Probe sequences | D** | Human miScript Primer for: let-7g-5p (Cat No MS00008337), let7i-5p (Cat No MS00003157), miR-21-5p (Cat No MS00009079), miR-22-3p (Cat No MS00003220), miR-29a-3p (Cat No MS00003262), miR-29b-3p (Cat No MS00006566), miR-29b-1-5p (Cat No MS00009289), miR-100-5p (Cat No MS00031234), miR-221-3p (Cat No MS00003857), miR-424-5p (Cat No MS00004186), miR-451a (Cat No MS00004242), miR-503-5p (Cat No MS00033838), miR-520e (Cat No MS00004536), miR-526b-3p (Cat No MS00031976), SNORD95 snoRNA (Cat No MS00033726), RNU6 snRNA (Cat No MS00033740) and miScript Universal Primer (miScript PCR kit, Qiagen)  Human FAM TaqMan Assays specific for ALK (assay ID Hs00608291_m1, Applied Biosystems)  Human FAM Double-Dye Assays specific for 18S, GAPDH, and UBC (Primer Design) |
| Location and identity of any modifications | **E** | none |
| Manufacturer of oligonucleotides | D | Qiagen  Applied Biosystems  Primer Design |
| Purification method | D |  |
| **qPCR PROTOCOL** |  |  |
| Complete reaction conditions | **E** | Expression of genes: we employed the geNORM Kit (Primerdesign) to choose the best reference genes (18S, GAPDH and UBC). ALK mRNA was analyzed in both NB cell lines and samples by using 25 ng of cDNA in two independent experiments, in duplicate, on EpGradient Realplex PCR System (Eppendorf), using Probe RealMasterMix (5PRIME). The comparative ΔΔCt method was then applied, normalizing Cq values of ALK on the geometric mean of the three housekeeping genes.  MiRNA expression: cDNAs from NB cell lines and tumor samples were analyzed with a miRNA-specific miScript Primer Assay and the miScript SYBR Green PCR Kit (Qiagen), containing the miScript Universal Primer and QuantiTect SYBR Green PCR Master Mix. |
| Reaction volume and amount of cDNA/DNA | **E** | Reaction volume 20 µl, 25 ng of cDNA. |
| Primer, (probe), Mg++ and dNTP concentrations | **E** | Commercial assay: 10x miScript primer assay; 20x TaqMan assay, 20x Double-Dye Assays, containing primers and probes  Mg^2+^ and dNTPs were included in the master mix, for genes we used Probe RealMasterMix (5PRIME) 5 mM Magnesium Acetate 0.4 mM dNTPs with dUTP final concentration; for miRNA we used QuantiTect SYBR Green PCR Master Mix (Qiagen) 2.5 mM MgCl_2_ final concentration and dUTP, which partially replaces dTTP |
| Polymerase identity and concentration | **E** | Probe RealMasterMix (5PRIME) containing HotMaster Taq DNA polymerase;  QuantiTect SYBR Green PCR Master Mix (Qiagen) containing HotStarTaq DNA Polymerase |
| Buffer/kit identity and manufacturer | **E** | For genes we used 2.5X Probe RealMasterMix (5PRIME) cat No F 2200700;  for miRNA we used 2X QuantiTect SYBR Green PCR Master Mix (Qiagen) Cat No./ID: 204143 |
| Exact chemical constitution of the buffer | D | For genes we used Probe RealMasterMix (5PRIME); for miRNAs we used QuantiTect SYBR Green PCR Master Mix (Qiagen) |
| Additives (SYBR Green I, DMSO, etc.) | **E** | For miRNAs SYBR Green I contained in the QuantiTect SYBR Green PCR Master Mix |
| Manufacturer of plates/tubes and catalog number | D | Twin.tec PCR plate 96, unskirted (Eppendorf, catalog number 0030133331) |
| Complete thermocycling parameters | **E** | For genes: enzyme activation (10 min at 95°C) and 2-step amplification (15s 95°C and 60s 60°C annealing/extension/data collection) for 40 cycles  for miRNA: enzyme activation (15 min at 95°C) and 3-steps amplification (15s 94°C, 30s 55°C annealing and 30s at 70°C extension/data collection) for 40 cycles, followed by a melting curve (ramping from 55°C to 95°C in 20 minutes) to control specificity. |
| Reaction setup (manual/robotic) | D | Manual |
| Manufacturer of qPCR instrument | **E** | EpGradient Realplex PCR System (Eppendorf) |
| **qPCR VALIDATION** |  |  |
| Evidence of optimisation (from gradients) | D | Not performed |
| Specificity (gel, sequence, melt, or digest) | **E** | Melting curve analysis (ramping from 55°C to 95°C in 20 minutes) for miRNA assays. |
| For SYBR Green I, Cq of the NTC | **E** | Not detected (Cq>40) |
| Standard curves with slope and y-intercept | **E** | ALK: slope -3.29, y-intercept 37.9  18S: slope -3.08, y-intercept 34.6  GAPDH: slope -3.20, y-intercept 35.4  UBC: slope -3.31, y-intercept 36.1 |
| PCR efficiency calculated from slope | **E** | ALK 1.01  18S: 1.11  GAPDH: 1.05  UBC:1.01 |
| Confidence interval for PCR efficiency or standard error | D |  |
| r2 of standard curve | **E** | ALK: 0.998  18S: 0.989  GAPDH: 0.999  UBC: 0.997 |
| Linear dynamic range | **E** | ALK Cq 24-35  18S: Cq 15-32  GAPDH: Cq 22-33  UBC: Cq 23-30 |
| Cq variation at lower limit | **E** | standard deviation for ALK: 0.47  standard deviation for 18S: 0.08  standard deviation for GAPDH: 0.05  standard deviation for UBC: 0.04 |
| Confidence intervals throughout range | D |  |
| Evidence for limit of detection | **E** | All sample Cqs were within the Linear dynamic range detected for target and reference genes |
| If multiplex, efficiency and LOD of each assay. | **E** | Not multiplexed |
| **DATA ANALYSIS** |  |  |
| qPCR analysis program (source, version) | **E** | RealPlex software v. 2.0 (Eppendorf, Hamburg, Germany) |
| Cq method determination | **E** | The threshold is used to specify Cq values of samples. The Cq value is the cycle in which the fluorescence signal intersects with the threshold. The threshold is determined using the Noise-band method: the threshold is specified so that it is significantly (10 times the standard deviation) above the noise of the baseline. The baseline is automatically calculated for every sample individually. (Derived from Mastercycler ep Realplex Instruction Manual) |
| Outlier identification and disposition | **E** | Runs were performed in triplicate. Single runs were excluded when the melting curve analysis revealed unintended amplification products melting. |
| Results of NTCs | **E** | Cq>40 |
| Justification of number and choice of reference genes | **E** | The geNORM Kit (Primerdesign) was employed to choose the best reference genes (18S, GAPDH and UBC). |
| Description of normalization method | **E** | For each cDNA, the duplicate Cq values were averaged, and normalized using reference genes, according the delta Cq method. |
| Number and concordance of biological replicates | D |  |
| Number and stage (RT or qPCR) of technical replicates | **E** | qPCR reactions were performed in duplicate. |
| Repeatability (intra-assay variation) | E | For each sample, standard deviation (SD) for the Cq variance between replicates has been used to express intra-assay variation. |
| Reproducibility (inter-assay variation, %CV) | D |  |
| Power analysis | D |  |
| Statistical methods for result significance | **E** | Non parametric Mann-Whitney, two tailed |
| Software (source, version) | E | RealPlex software v. 2.0 (Eppendorf, Hamburg, Germany); Microsoft Excel |
| Cq or raw data submission using RDML | **D** | No |
|  |  |  |
